# Evaluating regional heritability mapping methods for identifying QTLs in a wild population of Soay sheep

**DOI:** 10.1038/s41437-025-00770-0

**Published:** 2025-05-23

**Authors:** Caelinn James, Josephine M. Pemberton, Pau Navarro, Sara Knott

**Affiliations:** 1 Institute of Ecology and Evolution, School of Biological Sciences, The University of Edinburgh, Edinburgh, UK; 2 Scotland’s Rural College (SRUC), The Roslin Institute Building, Midlothian, UK; 3 The Roslin Institute and Royal (Dick) School of Veterinary Studies, The University of Edinburgh, Midlothian, UK; 4 MRC Human Genetics Unit, Institute of Genetics and Cancer, The University of Edinburgh, Edinburgh, UK

**Keywords:** Quantitative trait loci, Quantitative trait

## Abstract

The study of complex traits and their genetic underpinnings is crucial for understanding the evolutionary processes and mechanisms that shape natural populations. Regional heritability mapping (RHM) is a method for estimating the heritability of genomic segments that may contain both common and rare variants affecting a complex trait. This research is important because it advances our ability to detect genetic loci that contribute to phenotypic variation, even those that might be missed by traditional methods such as genome-wide association studies (GWAS). Here, we compare three RHM methods: SNP-RHM, which uses genomic relationship matrices (GRMs) based on SNP genotypes; Hap-RHM, which utilizes GRMs based on haplotypes; and SNHap-RHM, which integrates both SNP-based and haplotype-based GRMs jointly. These methods were applied to data from a wild population of sheep, focusing on the analysis of eleven polygenic traits. The results were compared with findings from previous GWAS to assess how RHM performed at identifying both known and novel associated loci. We found that while the inclusion of the regional matrix did not account for significant variation in all regions associated with trait variation as identified by GWAS, it did uncover several regions that were not previously linked to trait variation. This suggests that RHM methods can provide additional insights into the genetic architecture of complex traits, highlighting regions of the genome that may be overlooked by GWAS alone. This study underscores the importance of using complementary approaches to fully understand the genetic basis of complex traits in natural populations.

## Introduction

Finding the exact causal variants influencing a polygenic trait is often challenging, due to both the number of variants involved and the low effect sizes of the causal variants (Boopathi 2013). Some approaches instead look for variants in linkage disequilibrium (LD) with the causal variants to identify regions of the genome in which the causal variants reside (these regions are referred to as quantitative trait loci—QTL) (Hirschhorn and Daly 2005). Whilst the variants uncovered during QTL identification may not directly affect the focal trait, they can serve as markers for the causal variants and help researchers to locate these casual variants or to understand the size of their effects. By using these approaches, researchers can gain important information about the genetic architecture of a trait, even if they cannot directly identify the causal variants without performing functional studies. Genome-wide association studies (GWAS) are commonly used to identify genotyped SNPs in LD with causal loci. However, GWAS have some limitations and challenges that prevent it from finding all the genetic factors that contribute to complex traits (Du et al. 2012). One of these limitations is the power of GWAS (Yang et al. 2010), which is the ability of GWAS to detect true SNP-trait associations. The power of GWAS depends on several factors, such as the sample size, the variant effect size and frequency and the LD between genotyped and causal SNPs (Yang et al. 2010).

To overcome the limitations of GWAS, especially when a trait is influenced by multiple independent effects and/or rare variants in a region, regional heritability mapping (RHM) methods have been developed (Nagamine et al. 2012; Shirali et al. 2018; Oppong et al. 2021). RHM is a technique that estimates the heritability of a trait that is explained by a specific region of the genome. To estimate the heritability of a region, RHM uses a genomic relationship matrix (GRM), which is a matrix that captures the genetic similarity between individuals based on the proportion of shared SNP genotypes in that region (Yang et al. 2010). RHM also corrects for the genetic similarity across the whole genome by fitting another GRM that includes all the SNPs in the genome (or a leave-one-chromosome-out (LOCO) GRM that excludes the chromosome where the region of interest is located) (Yang et al. 2010). By comparing the model fit to a null model that does not fit the regional GRM (rGRM), RHM can identify regions that contain causal variants for the trait and by using the variance estimate for the rGRM, RHM can estimate how much heritability that region contributes.

RHM can be performed using different types of rGRMs and region sizes, depending on the assumptions and goals of the analysis. There are three main types of RHM that have been proposed. The first type is SNP-RHM, which uses rGRMs that are based on the sharing of SNP alleles across a region. The regions are usually defined as windows that contain a fixed number of SNPs (Nagamine et al. 2012). SNP-RHM aims to identify regions with multiple SNPs that are in LD with the multiple causal variants that have too small an effect on the trait individually to be detected by GWAS. However, SNP-RHM only captures effects associated with genotyped SNPs and only captures additive variance. The second type is Hap-RHM, which uses rGRMs that are based on the sharing of haplotype alleles across a region. The regions are defined as haplotype blocks (Shirali et al. 2018). Hap-RHM aims to identify regions where the causal variant is in LD with the haplotype allele, but not necessarily with any specific genotyped SNPs, which allows for detection of variance that is not captured by genotyped SNPs. This method can capture the effect of rare causal variants due to rare haplotype alleles being more likely to be in LD with rare variants than individual, genotyped SNPs. In addition, haplotype effects may reflect the interaction effects of closely linked causal variants (epistasis). The third method, SNHap-RHM, simultaneously fits two rGRMs: one SNP-based and one haplotype-based, and defines regions as haplotype blocks (Oppong et al. 2021). This combines the advantages of both SNP-RHM and Hap-RHM to increase power to detect regions containing variants influencing the phenotype. On occasions where SNP-RHM and Hap-RHM can detect genetic variance in the same haplotype block, SNHap-RHM can also be used to give more insight into the underlying genetic architecture.

Here, we evaluate the three RHM methods for their ability to identify regions containing potentially causal loci in a sample of wild Soay sheep. In this study, we analysed 11 polygenic morphometric traits in the Soay sheep population using RHM. These traits include the same traits measured at different ages, as they are affected by different non-genetic factors (and potentially different genetic factors) and vary in heritability across different stages of life. Despite using various methods to search for the genetic variants that affect these traits, such as GWAS (Bérénos et al. 2015; James et al. 2022), genomic prediction (Ashraf et al. 2021) and chromosome partitioning (Bérénos et al. 2015), most of the genetic variation in these traits remains unexplained by the genotyped and imputed SNPs. Moreover, for some of these traits, there are no SNPs that show significant association with the trait variation to date.

This paper aims to evaluate the applicability of RHM methods for Soay sheep data, considering the smaller sample sizes, lower density SNP data and higher potential for missing data compared to human datasets. To do this, we compare RHM results with GWAS to assess RHM’s ability to recover known associations and identify new ones. Additionally, this paper uses the results of the RHM analyses to enhance the understanding of the genetic architecture of focal traits and identifying potential causal genes based on functional data.

## Methods

### Phenotypic data

The Soay sheep (*Ovis aries*) is a primitive breed of sheep that lives on the St. Kilda archipelago, a small group of islands off the west coast of Scotland. Since 1985, a long-term, individual-based study has been conducted on the population residing on the island of Hirta, the largest of the islands (Clutton-Brock and Pemberton 2003). Each individual is sampled for DNA analysis and ear-tagged when it is first captured (usually within 10 days of birth) so that it can be re-identified later. The study involves regular recaptures to measure various traits throughout an individual’s life, and collection and measurement of skeletal remains after death.

We focused on 11 age-specific morphometric traits which have been repeatedly analysed by different approaches and are known to be polygenic (Bérénos et al. 2015; Ashraf et al. 2021; Hunter et al. 2022; James et al. 2022) (see Table 1 for the number of individuals and records per trait). We analysed these traits separately by age class (neonate, lamb and adult). Birth weight was the only trait analysed in neonates, defined as individuals who were caught and weighed between two and 10 days after birth. In August, lambs (aged ~4 months) and adults were caught and measured for weight, foreleg length and hindleg length. Due to adults being recaptured across multiple years, the adult live traits included repeated measurements. Metacarpal length and jaw length were measured from the skeletons after death. We classified ‘lambs’ as individuals who had live trait data recorded in the August of their birth year, or who died before 14 months of age for post mortem measures. We classified ‘adults’ as individuals who had live trait data recorded at least 2 years after birth, or who died after 26 months of age for post mortem measures. Birth and August weights are recorded to the nearest 0.1 kg, whilst the length traits are measured to the nearest mm (Beraldi et al. 2007). We did not analyse yearlings due to low sample size.

**Table 1:** Number of individuals and records, fixed and random effects fitted in each trait and age class model during RHM pre-correction, alongside the LOCO GRM.

Age	Trait	Number of individuals	Number of records	Fixed effects	Random effects
Neonate	Birth weight	2975	2975	Sex	Year of birth
				Litter size	Mother ID
				Population size year before birth	
				Age of mother (quadratic)	
				Ordinal date of birth	
				Age (days)	
Lamb	Weight	2424	2424	Sex	Year of birth
				Litter size	Mother ID
				Population size	
				Age (days)	
	Foreleg	2512	2512	Sex	Year of birth
				Litter size	Mother ID
				Population size	
				Age (days)	
	Hindleg	2577	2577	Sex	Year of birth
				Litter size	Mother ID
				Population size	
				Age (days)	
	Metacarpal	2117	2117	Sex	Year of birth
				Litter size	Mother ID
				Age at death (months)	
	Jaw	2172	2172	Sex	Year of birth
				Litter size	Mother ID
				Age at death (months)	
Adult	Weight	2092	3860	Sex	Year of capture
				Population size	Permanent environment
				Age (years)	
	Foreleg	1936	3594	Sex	Year of capture
				Population size	Permanent environment
				Age (years)	
	Hindleg	2027	3481	Sex	Year of capture
				Population size	Permanent environment
				Age (years)	
	Metacarpal	987	987	Sex	Year of birth
				Age at death (years)	
	Jaw	1057	1057	Sex	Year of birth
				Age at death (years)	

### Genetic data

8557 sheep have been genotyped on the Ovine SNP50 Illumina BeadChip, of which 38,130 SNPs are autosomal and polymorphic in the population. 188 individuals have additionally been genotyped on the Ovine Infinium HD SNP BeadChip which genotypes 600 K SNPs; these individuals were specifically selected to maximise the genetic diversity represented in the full population. This allowed for imputation of the remaining genotyped individuals to this higher density. AlphaImpute v1.98 (Hickey et al. 2012) was used for the imputation as it combines shared haplotype and pedigree information to increase imputation accuracy (see Stoffel et al. 2021 for details on our imputation). Genotypes with a probability of <0.99 were excluded, resulting in 419,281 autosomal SNPs remaining for 8557 individuals (4035 females, 4452 males). Cross-validation with the 50 K SNP genotype data gave a concordance rate of 0.995. Imputed genotype ‘hard’ calls were used instead of genotype probabilities in the analyses detailed in this manuscript. We have previously shown that imputation does not affect whole-genome heritability estimates for these traits in this population (James et al. 2022).

Locus positions for both sets of genetic data were based on the OAR_v3.1 genome assembly. Phased data is required for Hap-RHM and SNHap-RHM; genotypes were phased using SHAPEIT v4.2 (Delaneau et al. 2019).

### Splitting the genome into regions

In this paper, we used three RHM methods: SNP-RHM, Hap-RHM and SNHap-RHM. To allow for direct comparisons of results, we used the same regions for each RHM method. Due to Hap-RHM and SNHap-RHM requiring regions to be defined as haplotype blocks, we used haplotype blocks for all three methods. Haplotype blocks were estimated with Plink v1.90’s—*blocks* command (Purcell et al. 2007; Purcell 2014) using the high-density imputed genotype data. All SNPs with a MAF higher than 0.01 were included when calculating haplotype block boundaries, and any gaps larger than 500 kb between consecutive SNPs were automatically considered to be haplotype block boundaries. Using a higher max kb threshold or lower MAF threshold did not alter the haplotype block boundaries estimated.

No haplotype block was allowed to have only one SNP, due to the SNP-based GRM and haplotype-based GRM being identical for such blocks, resulting in the SNP-RHM and Hap-RHM methods therefore being identical and SNHap-RHM fitting two identical (and thus confounding) GRMs. Any block containing only one SNP was therefore omitted from the analysis.

Blocks were determined using all 8557 individuals with imputed genotypes to ensure consistency across phenotypes.

### Pre-correction of phenotypes

To perform the RHM analyses, we used DISSECT (Canela-Xandri et al. 2015) as it has the ability to generate the haplotype-based GRMs. However, pre-correction is a necessary step when performing RHM with DISSECT due to DISSECT being unable to fit the necessary fixed and random effects during the RHM step.

Prior to RHM, the traits were pre-corrected to account for genome-wide genetic diversity by fitting LOCO GRMs, which are constructed from all autosomes with the exception of one chromosome (Yang et al. 2014). The LOCO GRMs were computed using DISSECT and the VanRaden 2 method (VanRaden 2008), with the genetic relationship between individuals *i* and *j* is computed as:

$${A}_{{ij}}=\frac{1}{N}\mathop{\sum }\limits_{k=1}^{N}\frac{\left({s}_{{ik}}-2{p}_{k}\right)\left({s}_{{jk}}-2{p}_{k}\right)}{2{p}_{k}\left(1-{p}_{k}\right)}$$

where *s*_*ik*_ is the number of copies of the reference allele for SNP *k* of the individual *i*, *p*_*k*_ is the frequency of the reference allele for the SNP *k*, and *N* is the number of SNPs.

We also fitted fixed and non-genetic random effects during pre-correction (see Table 1 for a full list of fixed and non-genetic random effects fitted). Pre-correction for the non-repeated measures traits was performed in DISSECT (Canela-Xandri et al. 2015) using the following model:

$${\rm{y}}={\rm{X}}{\rm{\beta }}+\sum _{r}{{\rm{Z}}}_{r}{{\rm{u}}}_{r}+{\rm{W}}{g}_{{LOCO}}+{\rm{\varepsilon }}$$

where *y* is the vector of phenotypic values; *X* is a design matrix linking individual records with the vector of fixed effects *β*, *Z*_*r*_ is an incidence matrix that relates a random effect to the individual records; *u*_*r*_ is the associated vector of non-genetic random effects; *g*_*LOCO*_ is the vector of additive genetic random effects from all autosomes except for that containing the focal region with *W* the incidence matrix linking individual phenotypes with the genetic effect; and *ε* is the vector of residuals. It is assumed that *g*_*LOCO*_ ~ *MVN*(0, *Mσ*_*gLOCO*_^2^), where *σ*_*gLOCO*_^*2*^ is the additive genetic variance explained by all autosomes except the excluded one, and *M* is the LOCO GRM.

As there are 26 autosomes in the sheep genome, we generated 26 LOCO GRMs—this in turn resulted in each trait having 26 pre-corrected phenotypes. The advantage of using LOCO GRMs over a single whole genome GRM (which would result in one pre-corrected phenotype per trait) is that when performing RHM, when a rGRM is fitted, we can use the pre-corrected phenotype that excluded the chromosome on which the region (the haplotype blocks generated in the previous subsection) is located, preventing the effect of the SNPs in that region from being fitted twice in the model.

The residual for each individual was then taken as the pre-corrected phenotype for RHM:

$${y}_{{pre}-{corrected}}={\rm{\varepsilon }}$$

Pre-correction for the three repeated measures traits (adult August weight, adult foreleg length and adult hindleg length) was performed using ASReml-R (version 4.1, Butler et al. 2017) using the same model as given above, and the mean of the residuals summed with the permanent environment effect for each individual was taken as the phenotype for RHM:

$${y}_{{pre}-{corrected}}=\overline{{pe}}+{\rm{\varepsilon }}$$

### Regional heritability mapping

RHM was performed using DISSECT (Canela-Xandri et al. 2015), which generates both the SNP-based and haplotype-based rGRMs and simultaneously performs SNP-RHM, Hap-RHM and SNHap-RHM for each region all in one step.

#### SNP-RHM

SNP-RHM aims to identify regions with multiple SNPs that are in LD with the multiple causal variants that have too small an effect on the trait individually to be detected by GWAS by fitting a rGRM based on the sharing of SNP alleles across a region.

SNP-RHM is performed using the following model:

$${y}_{{pre}-{corrected}}={\rm{W}}{r}_{{SNP}}+{\rm{e}}$$

where *y*_*pre-corrected*_ is the vector of pre-corrected phenotypic values, *r*_*SNP*_ is the vector of individual additive genetic random effects from all SNPs contained within the focal haplotype block and *e* is the vector of residuals. It is assumed that r_SNP_ ~ *MVN*(0, *Mσ*_*rSNP*_^2^), where *σ*_*rSNP*_^*2*^ is the additive genetic variance from all SNPs in the haplotype block and *M* is the GRM. The GRMs were computed using DISSECT (Canela-Xandri et al. 2015). The SNP-based GRMs were calculated using the same method as the LOCO GRMs, except they were constructed from the SNPs located in the focal haplotype block.

#### Hap-RHM

Hap-RHM aims to identify regions where the causal variant is in LD with the haplotype allele, but not necessarily with any specific genotyped SNPs. This allows for detection of variance that is not captured by genotyped SNPs and can capture the effect of rare causal variants due to rare haplotype alleles being more likely to be in LD with rare variants than individual, genotyped SNPs. In addition, haplotype effects may reflect the interaction effects of closely linked causal variants.

Hap-RHM is performed using the following model:

$${y}_{{pre}-{corrected}}={\rm{W}}{r}_{{Hap}}+{\rm{e}}$$

where *y*_*pre-corrected*_ is the vector of pre-corrected phenotypic values, *r*_*Hap*_ is the vector of individual additive genetic random effects from the haplotype alleles for the focal haplotype block and *e* is the vector of residuals. It is assumed that r_Hap_ ~ *MVN*(0, *Hσ*_*rHap*_^2^), where *σ*_*rHap*_^*2*^ is the additive genetic variance from the haplotype alleles and *H* is the GRM. The haplotype-based GRMs were computed using DISSECT (Canela-Xandri et al. 2015), and the genetic relationship individuals *i* and *j* is calculated as follows:

$${H}_{{ij}}=\frac{1}{h}\mathop{\sum }\limits_{k=1}^{h}\frac{\left({d}_{{ik}}-2{p}_{k}\right)\left({d}_{{jk}}-2{p}_{k}\right)}{2{p}_{k}\left(1-{p}_{k}\right)}$$

where *d*_*ik*_ is the diplotype code (coded as the number of copies of haplotype *k* for individual *i* and takes the values 0, 1, and 2, *pk* is the frequency of haplotype *k* and *h* is the number of haplotypes in the region (see Oppong et al. 2021 for further information and examples).

#### SNHap-RHM

SNHap-RHM simultaneously fits both a SNP-based rGRM and a haplotype-based rGRM, which combines the advantages of both SNP-RHM and Hap-RHM to increase power to detect regions containing variants influencing the phenotype. On occasions where SNP-RHM and Hap-RHM can detect genetic variance in the same haplotype block, SNHap-RHM can also be used to give more insight into the underlying genetic architecture.

SNHap-RHM is performed using the following model:

$${y}_{{pre}-{corrected}}={\rm{W}}{r}_{{SNP}}+{\rm{W}}{r}_{{Hap}}+{\rm{e}}$$

where *y*_*pre-corrected*_ is the vector of pre-corrected phenotypic values, *r*_*SNP*_ is the vector of individual additive genetic random effects from all SNPs contained within the focal haplotype block and *r*_*Hap*_ is the vector of individual additive genetic random effects from the haplotype alleles for the focal haplotype block and *e* is the vector of residuals. It is assumed that r_SNP_ ~ *MVN*(0, *Mσ*_*rSNP*_^2^) and r_Hap_ ~ *MVN*(0*, Hσ*_*rHap*_^2^), where *σ*_*rSNP*_^*2*^ is the additive genetic variance from all SNPs in the haplotype block, *σ*_*rHap*_^*2*^ is the additive genetic variance from the haplotype alleles and *M* and H are the respective GRMs. The GRMs were computed using DISSECT (Canela-Xandri et al. 2015). The rGRMs were calculated as for SNP-RHM and Hap-RHM, respectively.

#### Null model and multiple testing

To test whether the regional heritability models explained significant variation for each region, we compared them against the null model:

$${y}_{{pre}-{corrected}}={\rm{e}}$$

using loglikelihood ratio testing (LRT).

We performed five comparisons; SNP-RHM, Hap-RHM and SNHap-RHM were all compared with the null model, and SNHap-RHM was additionally compared to each of SNP-RHM and Hap-RHM individually. LRTs were performed with 1 degree of freedom, with the exception of the comparison of SNHap-RHM to the null model, which was performed with 2 degrees of freedom. *P* values were calculated as 0.5× the *p* value of a chi-squared distribution with one degree of freedom for the 1 degree of freedom tests. For the 2 degrees of freedom tests, the *p* values were calculated as 0.25× the *p* value of a chi-squared distribution with two degrees of freedom plus 0.5× the *p* value of a chi-squared distribution with one degree of freedom (Self and Liang 1987). To account for multiple testing, model fit was considered to be significantly improved if the resulting *p* value was less than 1.04e^−06^ (0.05 divided by 48,125, the total number of haplotype blocks).

### Comparison with GWAS

To determine how well the different RHM methods detected previously discovered loci, we identified which haplotype blocks contained the top SNP from each peak significantly associated with phenotypic variation for each trait when performing GWAS. GWAS and conditional GWAS analysis has recently been performed using the high density genotype data (James et al. 2022), so we used the results from that analysis. The significance threshold used in the GWAS analysis was 1.03e^−06^ (0.05/48,635) (James et al. 2022), which accounted for multiple testing using the SimpleM method (Gao et al. 2008). This method accounts for LD between markers in order to calculate the effective number of independent tests. We also compared the proportion of genetic variance explained by the regions for which model fit was significantly improved for each of the RHM methods against the proportion of genetic variance explained by previous GWAS results. In loci with multiple significant regions, only the region with highest heritability was included in the calculation.

### Identification of candidate genes

We extracted a list of genes overlapping any haplotype block for which model fit was improved by at least one RHM model, using the R biomaRt package (Durinck et al. 2005; Durinck et al. 2009) from the OAR_v3.1 genome assembly. Each gene was then reviewed against the Ensembl (Howe et al. 2020) and NCBI Gene (Bethesda (MD): National Library of Medicine (US) 2004–2023) databases to examine expression and functional annotations. Human and mouse orthologues were also used to characterise gene function due to the high level of genetic annotation in these two species.

## Results

### Soay sheep haplotype blocks

Setting the maximum kb between any two variants within the same haplotype block to 500 Kb and the minimum minor allele frequency (MAF) for variants to be considered to 0.01 resulted in 48,125 haplotype blocks being estimated across the 26 Soay sheep autosomes. The maximum number of SNPs in a given haplotype block was 111, the minimum was 2 (as blocks with one SNP were omitted), and the average number of SNPs per haplotype was 8.19. 75% of haplotype blocks contained 10 or less SNPs, and 99% of blocks contained 50 or less. Additional information about the haplotype blocks can be found in the Supplementary Text.

### Comparison of RHM

A summary of results for the RHM analyses are shown in Tables 2 and 3, whilst detailed results are shown in Supplementary Tables 1–10 For ease of reporting, we have grouped the traits into the following; birth weight and lamb August weight, lamb leg length traits, lamb jaw length, adult August weight, adult leg length traits, and adult jaw length. To assess whether RHM is capable of identifying both previously associated loci and novel loci in comparison to GWAS, we compared the results to James et al. (2022).

**Table 2:** Number of haplotype blocks for which inclusion of regional GRMs improved model fit.

Trait	SNP-RHM	Hap-RHM	SNHap-RHM	SNHap-RHM vs Hap-RHM	SNHap-RHM vs SNP-RHM
Birth weight	0	0	0	0	0
Lamb August weight	0	0	0	0	0
Lamb foreleg length	0	2	0	0	0
Lamb hindleg length	0	2	0	0	0
Lamb metacarpal length	40	25	30	4	2
Lamb jaw length	0	5	0	0	2
Adult August weight	0	83	35	0	56
Adult foreleg length	0	6	6	0	6
Adult hindleg length	4	25	14	0	17
Adult metacarpal length	19	12	16	0	0
Adult jaw length	0	6	2	0	3

*SNP-RHM* column compares the SNP-RHM model against the null model to see if the inclusion of the regional SNP GRM improves model fit. *Hap-RHM* column compares the Hap-RHM model against the null model to see if the inclusion of the regional haplotype GRM improves model fit. *SNHap-RHM* column compares the SNHap-RHM model against the null model to see if the simultaneous inclusion of both the regional SNP GRM and the regional haplotype GRM improves model fit. *SNHap-RHM vs Hap-RHM* column compares the SNHap-RHM model against the Hap-RHM model to see if the additional inclusion of the regional SNP GRM improves model fit. *SNHap-RHM vs SNP-RHM* column compares the SNHap-RHM model against the SNP-RHM model to see if the additional inclusion of the regional haplotype GRM improves model fit.

**Table 3:** Percentage of genetic variance explained for each trait by each RHM method, and previous GWAS analyses (James et al. 2022).

Trait	Percentage of genetic variance explained by SNP-RHM	Percentage of genetic variance explained by Hap-RHM	Percentage of genetic variance explained by SNHap-RHM	Percentage of genetic variance explained by GWAS
Lamb foreleg length	0.00%	6.84%	0.00%	1.17%
Lamb hindleg length	0.00%	10.19%	0.00%	1.48%
Lamb metacarpal length	56.59%	13.64%	77.16%	5.14%
Lamb jaw length	0.00%	10.05%	1.79%	0.00%
Adult August weight	0.00%	46.10%	63.33%	9.31%
Adult foreleg length	0.00%	4.13%	5.29%	9.49%
Adult hindleg length	0.17%	2.68%	2.71%	5.24%
Adult metacarpal length	27.09%	5.24%	27.58%	8.03%
Adult jaw length	0.00%	7.84%	9.24%	2.39%

Birth weight and lamb August weight have not been included, as no genetic variance has been explained by any of these methods.

#### Birth weight and lamb August weight

None of the RHM models significantly improved model fit for any haplotype blocks for either birth weight or lamb August weight, meaning that no regions of the genome were found to significantly explain additional genetic variance not accounted for during pre-correction (see ‘Methods’).

#### Lamb leg length traits

**Fig. 1:**
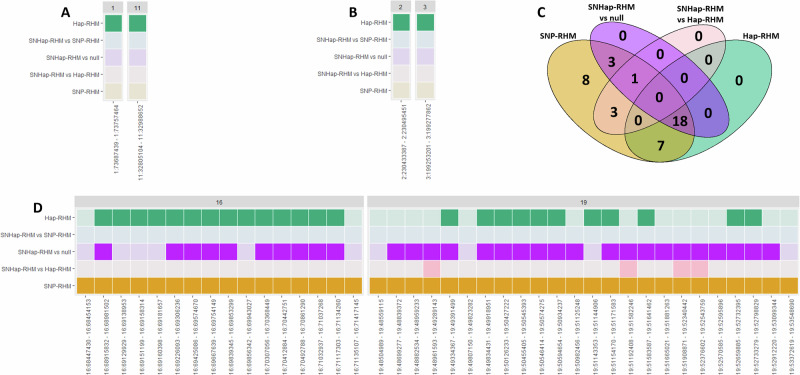
Distribution of haplotype blocks for which at least one RHM model significantly improved model fit for the three lamb leg length traits. **A** Heatmap indicating which RHM models significantly improved model fit for haplotype blocks across the genome for lamb foreleg length. **B** Heatmap indicating which RHM models significantly improved model fit for haplotype blocks across the genome for lamb hindleg length. **C** Venn diagram showing the number of haplotype blocks for which each RHM model showed significantly improved model fit for lamb metacarpal length, including the overlaps of blocks for which model fit was significantly improved by two or more models. **D** Heatmap indicating which RHM models significantly improved model fit for haplotype blocks across the genome for lamb metacarpal length.

For lamb foreleg length and lamb hindleg length, Hap-RHM was the only model which significantly improved model fit in comparison to the null model. Improved model fit was shown for one haplotype block on chromosome 1 and one on chromosome 11 for lamb foreleg length (Fig. 1A), and one on chromosome 2 and chromosome 3 for lamb hindleg length (Fig. 1B) (Supplementary Tables 1–3). All four of these blocks are novel associations, as they do not contain SNPs that have previously been found to be associated with any lamb leg length trait. The regions for which Hap-RHM significantly improved model fit explained 6.84% of the total genetic variance for lamb foreleg length and 10.19% of the total genetic variance for lamb hindleg length (Table 3); in comparison, the independently associated SNPs from GWAS analyses explained 1.17% and 1.48% for each trait respectively. Seven genes overlapped with these four haplotype blocks; however, none have a clear link to leg length, skeletal size or growth (Supplementary Table 1).

For lamb metacarpal length, model fit was significantly improved for a total of 40 haplotype blocks across chromosomes 16 and 19 when using at least one RHM method. SNP-RHM improved model fit for all of these blocks, and 30 blocks also showed improved model fit for at least one other model, though no blocks showed increased model fit for SNHap-RHM when compared to SNP-RHM (Fig. 1C, Supplementary Tables 1 and 4).

On chromosome 16, there were 16 haplotype blocks that significantly improved model fit using SNP-RHM. Out of these, 14 blocks showed improved model fit using Hap-RHM, 10 of which also showed improved model fit by SNHap-RHM when compared to the null model (Fig. 1D, Supplementary Tables 1 and 4). On chromosome 19, there were 24 haplotype blocks for which SNP-RHM significantly improved model fit, of which 11 also showed increased model fit for Hap-RHM, 20 showed increased model fit for SNHap-RHM when compared to the null model, and four showed increased model fit for SNHap-RHM when compared to Hap-RHM. (Fig. 1D, Supplementary Tables 1 and 4).

In total, SNP-RHM explained 56.59% of the total genetic variance for lamb metacarpal length, Hap-RHM explained 13.64%, and SNHap-RHM explained 77.16% (Table 3). In comparison, previous GWAS results explained 5.14% of the total genetic variance.

Previous GWAS have found significant associations between lamb metacarpal length and SNPs on chromosomes 16 and 19; the haplotype block containing these SNPs on chromosome 16 showed significantly improved model fit for SNP-RHM, Hap-RHM and SNHap-RHM compared to the null model, whilst the block containing these SNPs on chromosome 19 showed significantly improved model fit for SNP-RHM, and SNHap-RHM when compared to the null model and to Hap-RHM.

165 genes overlapped the haplotype blocks for which model fit was significantly improved by at least one RHM method; of these, six had potential links to leg length and skeletal growth (Table 4, Supplementary Table 1).

**Table 4 -:** Potential candidate genes for future analyses.

Trait	Models with improved fit	Chromosome	Haplotype or SNP location(s) (bp)	Gene	Functional annotation
Adult August weight	Hap-RHM, SNHap-RHM vs null, SNHap-RHM vs SNP-RHM	1	40773086–40841455, 40847219–40872161	LEPR	Codes for the leptin receptor, an adipocyte-specific hormone that regulates body weight. Repeatedly associated with body weight and physiological factors contributing to body weight variation across multiple species (Chagnon et al. 1999; Yiannakouris et al. 2001; Israel and Chua 2010; Ros-Freixedes et al. 2016; Solé et al. 2021), including sheep (Macé et al. 2022).
Adult August weight	Hap-RHM	1	95596363–95600207	TBX15	Identified as a potential causal gene for birth weight in Barki sheep (Abousoliman et al. 2021). Found to be a master transcriptional regulator in human adipose tissue and has downstream effects on abdominal obesity (Pan et al. 2021), correlates with BMI and hip-to-waist ratio in humans (Heid et al. 2010) and TBX15 knock-out mice are shown to have increased body weight gain in comparison to control mice (Sun et al. 2019).
Adult August weight	Hap-RHM, SNHap-RHM vs null, SNHap-RHM vs SNP-RHM	2	38080434–38081124	EPHX2	Linked with obesity in humans (Khadir et al. 2020) and affects insulin sensitivity in both rodents and humans (Luther and Brown 2016).
Adult hindleg	Hap-RHM	6	103234182–103331382	EVC2	Humans: his gene encodes a protein that functions in bone formation and skeletal development. Mutations in this gene cause Ellis-van Creveld syndrome (characteristics include small stature and short limbs) (Baujat and Le Merrer 2007) and Weyers acrofacial dysostosis (characteristics include short limbs) (Ye et al. 2006). Deletion within EVC2 causes chondrodysplastic (short-legged) dwarfism in cattle (Murgiano et al. 2014).
Adult foreleg length Adult hindleg length	Hap-RHM, SNHap-RHM vs null, SNHap-RHM vs SNP-RHM	12	1315546–1498521	PPP1R15B	Missense mutation in PPP1R15B causes a disease in humans resulting in short stature (Abdulkarim et al. 2015)
Adult August weight	Hap-RHM, SNHap-RHM vs null, SNHap-RHM vs SNP-RHM	12	31886985–32003794	SDCCAG8	Mutations result in Bardet-Biedl Syndrome (characterisation includes obesity) in humans (Schaefer et al. 2011). Associated with obesity in human children and adolescents (Scherag et al. 2012)
Lamb jaw length	Hap-RHM, SNHap-RHM vs SNP-RHM	13	53300575–53763103	LOC101112800	Expressed in mouse lower jaw mesenchyme (Diez-Roux et al. 2011)
Lamb metacarpal length	SNP-RHM, SNHap-RHM vs null	19	48699277–48839372	POC1A	Mutations in this gene result in SOFT syndrome (characterisation includes short stature) in humans (Min Ko et al. 2016).
Lamb metacarpal length	SNP-RHM, Hap-RHM, SNHap-RHM vs null	19	50594654–50934237	TCTA	Induces osteoclastogenesis (Kotake et al. 2009)
Lamb metacarpal length	SNP-RHM, Hap-RHM, SNHap-RHM vs null	19	50594654–50934237	RHOA	Promotes osteoclastogenesis (Wang et al. 2023). Inhibition of RHOA induces chondrogenesis in chick limbs (Kim et al. 2012). Inhibits bone formation by suppressing IGF1 (Negishi-Koga et al. 2011).
Lamb metacarpal length	SNHap-RHM vs null, SNHap-RHM vs Hap-RHM, SNHap-RHM vs SNP-RHM	19	51192408–51582246	PLXNB1	Involved in negative regulation of osteoblast proliferation. Activates RHOA. (Negishi-Koga et al. 2011)
Lamb metacarpal length	SNHap-RHM vs null, SNHap-RHM vs Hap-RHM, SNHap-RHM vs SNP-RHM	19	52376602–52543759	PTH1R	Previously identified as potential causal gene for multiple leg length measures in Soay sheep via GWAS (James et al. 2022). Involved in osteoblast development in mice (Qiu et al. 2015), associated with skeletal disorders such as EKNS (Duchatelet et al. 2005), JMC and BLC (Schipani and Provot 2003) in humans.
Adult metacarpal length	SNHap-RHM vs null				
Lamb metacarpal length	SNP-RHM	19	53372619–53548690	LIMD1	Influences osteoblast differentiation and function in mice (Luderer et al. 2008)

From left to right: associated trait, method that resulted in the gene being identified, chromosome, haplotype block, gene name, and evidence for association in sheep and other species.

#### Lamb jaw length

**Fig. 2:**
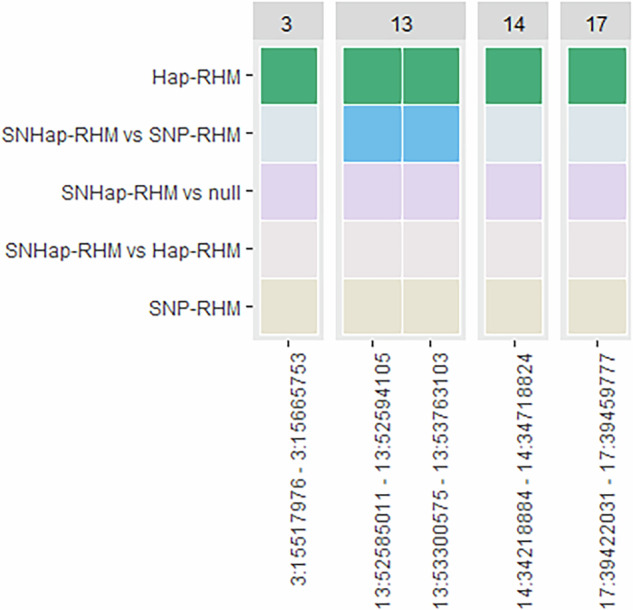
Distribution of haplotype blocks showing increased model fit for lamb jaw length. Heatmap indicating which RHM models significantly improved model fit for haplotype blocks across the genome for lamb jaw length.

For lamb jaw length, model fit was significantly improved for five haplotype blocks for at least one RHM method; one each on chromosomes 3, 14 and 17 and two blocks on chromosome 13. Hap-RHM was shown to improve model fit for all five of these blocks, and model fit for the two blocks on chromosome 13 was also improved by SNHap-RHM when compared to SNP-RHM (Fig. 2, Supplementary Tables 1 and 5). All five of these blocks are novel associations; no previous associations have been found for lamb jaw length. In total, Hap-RHM explained 10.05% of the total genetic variance, whilst SNHap-RHM explained 1.79%.

50 genes overlapped these five blocks; only one of these genes has a potential association with jaw length (Table 4, Supplementary Table 1).

#### Adult August weight

**Fig. 3:**
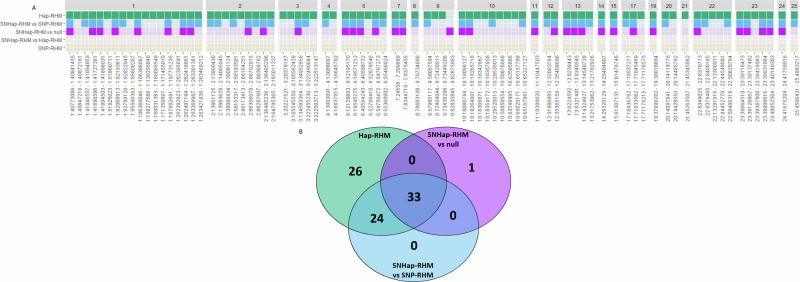
Distribution of haplotype blocks for which at least one RHM model significantly improved model fit for adult August weight. **A** Heatmap indicating which RHM models significantly improved model fit for haplotype blocks across the genome for adult August weight. **B** Venn diagram showing the number of haplotype blocks for which each RHM model showed significantly improved model fit for adult August weight, including the overlaps of blocks for which model fit was significantly improved by two or more models.

For adult August weight, model fit was significantly improved for 84 haplotype blocks over 22 chromosomes (Fig. 3A, Supplementary Tables 1 and 6). For 83 of these blocks, model fit was improved when using Hap-RHM, of which 57 blocks also showed improved model fit when using SNHap-RHM when compared to SNP-RHM, and 33 of those blocks also showed improved model fit when using SNHap-RHM when compared to the null model. The final block only showed significant improvement in model fit for SNHap-RHM when compared to the null model (Fig. 3B, Supplementary Tables 1 and 6). None of these blocks overlapped with previous GWAS associations for adult August weight (Supplementary Table 11).

Hap-RHM explained 46.10% of the total genetic variance for adult August weight, whilst SNHap-RHM explained 63.33% (Table 3). In comparison, previous GWAS results explained 9.31% of the total genetic variance.

86 genes overlapped these 84 blocks; of these four had associations with body weight and obesity (Table 4, Supplementary Table 1).

#### Adult leg length traits

**Fig. 4:**
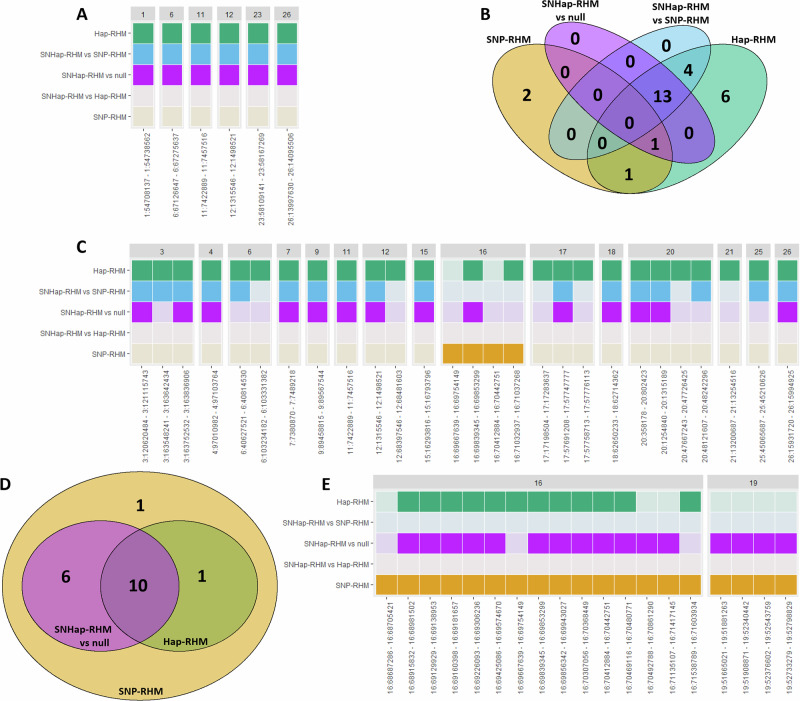
Distribution of haplotype blocks for which at least one RHM model significantly improved model fit for the three adult leg length traits. **A** Heatmap indicating which RHM models significantly improved model fit for haplotype blocks across the genome for adult foreleg length. **B** Heatmap indicating which RHM models significantly improved model fit for haplotype blocks across the genome for adult metacarpal length. **C** Heatmap indicating which RHM models significantly improved model fit for haplotype blocks across the genome for adult hindleg length. **D** Venn diagram showing the number of haplotype blocks for which each RHM model showed significantly improved model fit for adult metacarpal length, including the overlaps of blocks for which model fit was significantly improved by two or more models. **E** Heatmap indicating which RHM models significantly improved model fit for haplotype blocks across the genome for adult metacarpal length.

For adult foreleg length, model fit was significantly improved for six haplotype blocks; one each on chromosomes 1, 6, 11, 12, 23 and 26 (Fig. 4A, Supplementary Tables 1 and 7). For all six blocks, the models that improved model fit were Hap-RHM, SNHap-RHM when compared to the null model and SNHap-RHM when compared to SNP-RHM. All of these blocks were novel associations, as they did not contain SNPs that had previously been associated with leg length in prior analyses. Hap-RHM explained 4.13% of the total genetic variance for adult foreleg length, whilst SNHap-RHM explained 5.29% (Table 3). In comparison, previous GWAS results explained 9.49% of the total genetic variance.

For adult hindleg length, model fit was significantly improved for 27 haplotype blocks. 25 of these haplotype blocks showed significant model fit improvement for Hap-RHM, with 17 of them also showing significant model improvement for SNHap-RHM when compared to SNP-RHM, and another two showing significant model improvement for SNP-RHM. The remaining two blocks only showed significant model fit improvement for SNP-RHM (Fig. 4B, Supplementary Tables 1 and 8). The 27 haplotype blocks were distributed across 15 different chromosomes, with the four blocks for which SNP-RHM improved model fit all located on chromosome 16. These four blocks were the only blocks on chromosome 16 that showed improved model fit (Fig. 4C, Supplementary Tables 1 and 8). One of these blocks on chromosome 16 contained SNPs that had previously been associated with adult hindleg length when performing GWAS. The remaining blocks were all novel associations. SNP-RHM explained 0.17% of the total genetic variance for adult hindleg length, Hap-RHM explained 2.68% and SNHap-RHM 2.71% (Table 3). In comparison, previous GWAS results explained 5.24% of the total genetic variance.

For adult metacarpal length, model fit was significantly improved for a total of 19 haplotype blocks across chromosomes 16 and 19 when using at least one RHM method. SNP-RHM improved model fit for all of these blocks, and 18 blocks also showed improved model fit for at least one other model, though no blocks showed increased model fit for SNHap-RHM when compared to either SNP-RHM or Hap-RHM (Fig. 4D, Supplementary Tables 1 and 9).

On chromosome 16, there were 15 haplotype blocks that significantly improved model fit using SNP-RHM. Out of these, 12 blocks showed improved model fit using Hap-RHM and 12 showed improved model fit by SNHap-RHM when compared to the null model, though the blocks for which these two models improved model fit were not all the same (Fig. 4E, Supplementary Tables 1 and 9). On chromosome 19, there were 4 haplotype blocks significantly improved model fit, and all four blocks showed improved model fit for both SNP-RHM and SNHap-RHM when compared to the null model (Fig. 4E, Supplementary Tables 1 and 9). One of these blocks contained SNPs that had previously been associated with adult metacarpal length when performing GWAS. SNP-RHM explained a total of 27.09% of the total genetic variance for adult metacarpal length, whilst Hap-RHM explained 5.24% and SNHap-RHM 27.58% (Table 3); in comparison, GWAS results explained 8.03%.

83 genes overlapped all of the blocks that showed improved model fit across the three adult leg length traits. Of these, three had potential links to bone length and skeletal growth; one overlapping a block associated with both adult foreleg and hindleg length, one overlapping a block associated with adult hindleg length, and one overlapping a block associated with adult metacarpal length. The potential causative gene associated with adult metacarpal length also overlapped haplotype blocks showing improved model fit for lamb metacarpal length (Table 4, Supplementary Table 1).

#### Adult jaw length

**Fig. 5:**
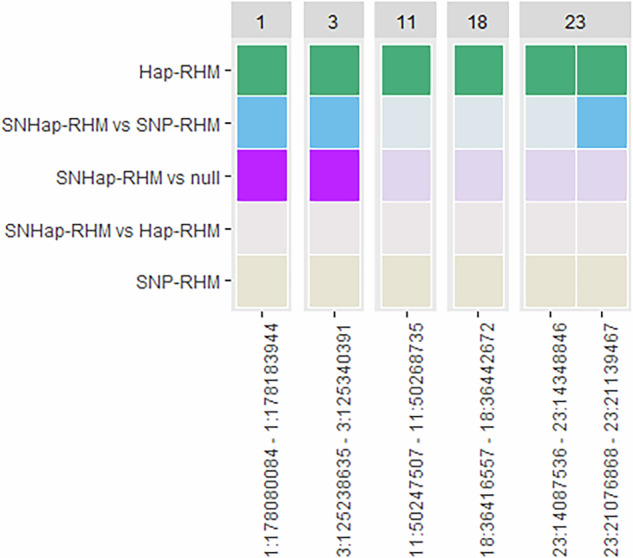
Distribution of haplotype blocks showing increased model fit for adult jaw length. Heatmap indicating which RHM models significantly improved model fit for haplotype blocks across the genome for adult jaw length.

For adult jaw length, model fit was significantly improved for six haplotype blocks for at least one RHM method; one each on chromosomes 1, 3, 11 and 18 and two blocks on chromosome 23. Hap-RHM was shown to improve model fit for all six of these blocks, whilst SNHap-RHM significantly improved model fit for the blocks on chromosomes 1 and 3, and one of the blocks on chromosome 23 when compared to SNP-RHM. The blocks on chromosomes 1 and 3 also showed improved model fit when using SNHap-RHM compared to the null model. (Fig. 5, Supplementary Tables 1 and 10). All six of these blocks are novel associations, as they do not contain SNPs that have previously been found to be associated with adult jaw length. Hap-RHM explained a total of 7.84% of the total genetic variance for adult jaw length, whilst SNHap-RHM explained 9.24% (Table 3). In comparison, previous GWAS results explained 2.39%.

Five genes overlapped these six blocks, though none had a clear association with jaw length or skeletal size (Supplementary Table 1).

## Discussion

### Summary of results

In total, there were 169 haplotype blocks for which model fit was improved for at least one trait by at least one RHM model. Novel block-trait associations were identified using at least one RHM method for all but four traits. In the case of birth weight and August lamb weight, RHM did not improve model fit for any haplotype blocks in comparison to the null model, whilst in the case of lamb and adult metacarpal length, RHM only improved model fit for previously identified QTL regions.

Across all haplotype blocks for which model fit was improved by at least one RHM method for at least one of the 11 focal traits, there are 351 genes overlapping these blocks. 91 of these genes are completely uncharacterised in sheep and classed as ‘novel genes’, and a further 14 genes are RNA genes (Supplementary Table 1). Of the 246 characterised protein coding genes, 13 genes had functional annotations that relate to the traits for which model fit was improved (Table 4). One of these genes is in a haplotype block associated with lamb jaw length, four in haplotype blocks associated with adult August weight, one associated with adult foreleg and adult hindleg length, one in a haplotype block associated only with adult hindleg length and eight in haplotype blocks associated with lamb metacarpal length (one of which was also associated with adult metacarpal length). One of these genes – *PTH1R* – was previously identified as a putative causal gene due to its functional data and proximity to top GWAS SNPs for multiple Soay sheep leg length measures (James et al. 2022).

### Comparison of RHM models and previous studies

We found that Hap-RHM improved model fit more often than SNP-RHM. This is due in part to the fact that Hap-RHM significantly improved model fit for more traits than SNP-RHM; Hap-RHM improved model fit for at least one block for all traits (with the exception of birth weight and lamb August weight), whilst SNP-RHM only improved model fit for lamb and adult metacarpal lengths. Hap-RHM also improved model fit more often than SNHap-RHM when SNHap-RHM was compared to either the null model or either of the single rGRM models.

Of the 11 traits, lamb August weight and lamb jaw length were the only two to have no previously associated genetic loci (Bérénos et al. 2014; James et al. 2022). Of the traits for which GWAS has previously identified SNP-trait associations, RHM only significantly improved model fit for blocks containing SNPs previously associated with lamb metacarpal length, adult hindleg length and adult metacarpal length on chromosomes 16 and 19. The three RHM models explained a higher proportion of the total genetic variance for each trait in comparison to independent significant SNPs from previous GWAS analyses, with the exception of adult foreleg length and adult hindleg length. It is worth noting that RHM failed to find known associations between chromosome 16 and adult foreleg length, and chromosome 19 and both adult foreleg length and adult hindleg length.

SNP-RHM has previously been performed in a smaller sample of this same population, focusing on only adult morphometric traits (Bérénos et al. 2015). 37 K autosomal SNPs were split into 150 SNP windows with a 75 SNP overlap. When comparing the results of Bérénos et al. (2015) to our results for the same traits, we find six regions for which SNP-RHM improved model fit for Bérénos et al. (2015) and at least one RHM method improved model fit in our own analyses; two regions on chromosome 1 and one region on chromosome 6 were associated with adult August weight, one region on chromosome 6 was associated with adult hindleg length, a region on chromosome 16 associated with adult hindleg and metacarpal length, and a region on chromosome 19 associated with adult metacarpal length.

Novel block-trait associations were identified using at least one RHM method for all but four traits – birth weight, lamb August weight, lamb metacarpal length and adult metacarpal length. In the case of the former two traits, RHM did not improve model fit for any haplotype blocks in comparison to the null model, whilst in the case of the latter two, RHM only significantly improved model fit in the same regions as previously identified QTL for these traits.

### Insights into genetic architecture

None of the haplotype blocks for which model fit was significantly improved for adult August weight were within 1 Mb of a previously identified GWAS association. Interestingly, neither SNP-RHM compared to the null model nor SNHap-RHM when compared to Hap-RHM improved model fit for any haplotype blocks for adult August weight. This suggests that the majority of genetic variance contributing to variation in adult August weight is not due to small effect causal variants in LD with genotyped SNPs, but instead due to rare SNPs in LD with rare haplotype alleles or due to multiple SNPs in the same region interacting epistatically. We have previously shown that family-associated non-additive genetic variance such as dominance and epistasis may be making up 37.1% of previous narrow-sense heritability estimations for this trait (James et al. 2023). This finding would be consistent with Hap-RHM detecting regions in which multiple variants are acting in an epistasic manner. We found four genes with functional data suggesting an association with adult August weight that overlapped with haplotype blocks for which model fit was significantly improved by at least one RHM method: *LEPR*, *TBX15, SDCCAG8* and *EPHX2*. For the blocks overlapping these genes, model fit was significantly improved by the presence of the haplotype GRM; Hap-RHM significantly improved model fit for all of the overlapping blocks, and SNHap-RHM significantly improved model fit when compared to the null model for the block overlapping *SDCCAG8* and to null model and SNP-RHM for the blocks overlapping *LEPR* and *EPHX2*. This may explain why these regions were not identified as being associated with adult August weight when performing GWAS (James et al. 2022), as the variance influencing adult August weight in those regions is likely due to specific haplotype alleles, rather than individual SNP effects.

The underlying causal variant on chromosome 16 influencing lamb metacarpal length is currently presumed to be the same variant influencing adult hindleg length and adult metacarpal length – the GWAS-significant SNPs on chromosome 16 for adult hindleg length and lamb metacarpal length are the same (James et al. 2022), adult hindleg and metacarpal length have been shown to have a genetic correlation of 0.827 (S.E. 0.232) (Bérénos et al. 2014), and SNP-leg trait associations in this region have been shown to be dependent on each other; when a SNP genotype from this region is fitted during conditional analyses, no new SNP associations appear in this region. We can therefore combine the RHM results for these three traits to characterise the architecture of genetic variance in this region. Whilst SNP-RHM significantly improved model fit for blocks on chromosome 16 that Hap-RHM did not, there were no blocks on chromosome 16 for which Hap-RHM improved model fit but SNP-RHM did not (Supplementary Tables 1, 4, 8 and 9). In fact, in the case of adult hindleg length, Hap-RHM did not improve model fit for any blocks on chromosome 16 (Supplementary Table 8). This suggests that the additive genetic variance being attributed to the rGRMs is due to individual SNP genotypes, rather than due to a specific haplotype allele. The haplotype block containing SNP s22142.1, (the SNP with the lowest *p* value for lamb metacarpal length and adult hindleg length when performing GWAS (James et al. 2022)) contains 17 SNPs and has 18 haplotype alleles in the population. The minor allele for s22142.1 appears in 3 haplotype alleles, with two of these haplotype alleles being relatively rare (each appearing on 17 chromosomes in the genotyped population).

The underlying causal variant on chromosome 19 influencing lamb metacarpal length is presumed to be the same variant influencing adult metacarpal length – whilst the SNP with the lowest *p* value when performing GWAS are different for these two traits, they still fall in the same haplotype block (Supplementary Table 11) and when the genotype of each of these SNPs is fitted during conditional analysis, no new SNP-trait associations appear (James et al. 2022). Similarly, we can combine the RHM results for both lamb metacarpal length and adult metacarpal length to characterise the underlying architecture. For both traits, model fit for the block containing the top GWAS SNPs was only significantly improved by SNP-RHM and SNHap-RHM when compared to the null model (and SNHap-RHM compared to Hap-RHM in the case of lamb metacarpal length). This suggests that this association is being driven by the SNP alleles in this region, rather than the haplotype alleles. The haplotype block containing the GWAS SNPs with the lowest *p* value has 37 SNPs and 52 haplotype alleles in the genotyped population. The minor alleles for each of these SNPs each appear in two haplotype alleles, with one haplotype allele containing both minor SNP alleles. The haplotype alleles each containing one of the minor SNP alleles for these SNPs were both rare in the population (appearing on one and 50 chromosomes in the population).

### Limitations of RHM

We have previously shown that pre-correcting for fixed and random effects reduces power of GWAS to detect variant-trait associations (James et al. 2022), as fitting covariates during analyses correctly propagates error throughout the analysis, reducing the chance of false positive results and increasing power by disentangling potential correlations. Pre-correction may therefore explain why we did not see the RHM methods improving model fit for all of the haplotype blocks containing previously identified variants. Currently pre-correction is a necessary step when performing RHM with DISSECT due to DISSECT being unable to fit all of the necessary fixed and random effects during RHM, and it is not possible to extract the haplotype-based GRMs from DISSECT to perform the analyses with different software. It would be interesting to rerun this analysis when suitable software is developed for single-step RHM, to determine whether single-step RHM improved model fit for all haplotype blocks containing significant GWAS associations. In addition, pre-correction may reduce the power to identify significant regions due to correlations between the LOCO GRM and the rGRM (as they are describing relatedness between the same set of related individuals). Using a LOCO GRM to pre-correct rather than a whole genome GRM should help alleviate this to some extent (as the SNPs used in the rGRM are therefore not used in the LOCO GRM), however this does not fully exclude the underlying issue. However, the presence of significant regions in our results suggests that this is not an issue for every region, and may only affect a small number.

Previous analyses using Hap-RHM and SNHap-RHM have proposed using the location of recombination hotspots (Shirali et al. 2018; Oppong et al. 2021), however this was not available for our population. Instead, we estimated haplotype blocks using Plink (Purcell et al. 2007; Purcell 2014). This gave us a total of 48,125 haplotype blocks containing at least two SNPs across the sheep genome. Previously, we have calculated the number of independent tests when performing GWAS using this same SNP density to be 48,635 (James et al. (2022)). Given how close these two figures are (especially as we excluded any haplotype blocks that only contained a single SNP), we are confident in the haplotype blocks that were generated and that identifying recombination hotspots was not necessary for our population.

We chose to exclude haplotype blocks with only one SNP, as the SNP-based and haplotype-based rGRMs would be identical. However, this means that any variance being contributed to the trait by these SNPs has been missed from our analyses. Ideally, SNP density and distribution should be such that each haplotype block has at least two SNPs, so that all haplotype blocks can be included in the analysis.

One limitation of RHM is the challenge of determining whether the identified associations are genuine or simply false positives. This uncertainty can stem from various factors, such as statistical noise or random chance due to the testing of multiple regions. Additionally, the regions flagged as significant may contain complex genetic interactions or overlapping effects, making it difficult to pinpoint the true cause of the association. Therefore, further studies such as functional analyses are required to separate the true associations from any potential false positives.

## Concluding remarks

Here, we have demonstrated that RHM methods are a useful tool for detecting regions that contribute genetic variation to traits in a wild population and complement other analyses such as GWAS. We found that Hap-RHM and SNHap-RHM improved model fit for more haplotype blocks than SNP-RHM, but all three can be used together to better characterise the underlying genetic architecture within a region. Using these methods, we detected multiple haplotype blocks that improved model fit with at least one RHM method. From these regions, we characterised the genetic regions influencing trait variation and identified 13 potential causal genes that have not previously been associated with variation in these traits in the Soay population.

## Supplementary information

Supplementary Material

## Data Availability

All scripts and data can be found at https://github.com/CaelinnJames/RegionalHeritabilityMapping_SoaySheep.
