# Supplementary material for: Evaluating regional heritability mapping methods for identifying QTLs in a wild population of Soay sheep
Source: Heredity (Edinb). 2025 May 23;134(6):374–86. doi: 10.1038/s41437-025-00770-0 (PMC12137591; doi:10.1038/s41437-025-00770-0)
Supplement: Supplementary file 1 — Supplementary Material [file 41437_2025_770_MOESM1_ESM.pdf]

Chromosome	Block	Block start (bp)	Block end (bp)	Block length (Kb)	SNPs	Haplotype alleles	Models	Genes	NCBI Gene name	Functional data
1	948	40,773,086	40,841,455	68.37	10	14	Adult August weight (Hap-RHM, SNHap-RHM vs null, SNHap-RHM vs SNP-RHM)	ENSOARG00000010394	LEPR	Receptor for leptin (an adipocyte-specific hormone that regulates body weight). Mutations in this gene have been associated with obesity and pituitary dysfunction
1	949	40,847,219	40,872,161	24.943	7	11	Adult August weight (Hap-RHM)	ENSOARG00000010394	LEPR	
1	956	41,046,557	41,094,853	48.297	11	22	Adult August weight (Hap-RHM, SNHap-RHM vs SNP-RHM)			
1	974	41,690,586	41,727,381	36.796	10	28	Adult August weight (Hap-RHM, SNHap-RHM vs null, SNHap-RHM vs SNP-RHM)			
1	982	41,894,503	41,896,031	1.529	3	5	Adult August weight (Hap-RHM, SNHap-RHM vs null, SNHap-RHM vs SNP-RHM)			
1	1227	51,825,023	51,860,711	35.689	10	35	Adult August weight (Hap-RHM)			
1	1284	54,708,137	54,738,562	30.426	8	15	Adult foreleg length (Hap-RHM, SNHap-RHM vs null, SNHap-RHM vs SNP-RHM)	ENSOARG00000013529	ADGRL4	
1	1717	73,687,439	73,757,464	70.026	14	23	Lamb foreleg length (Hap-RHM)	ENSOARG00000017549	DPYD	
1	1908	82,068,913	82,193,611	124.699	19	29	Adult August weight (Hap-RHM, SNHap-RHM vs null, SNHap-RHM vs SNP-RHM)			
1	2203	92,789,120	92,822,945	33.826	9	21	Adult August weight (Hap-RHM, SNHap-RHM vs SNP-RHM)			
1	2294	95,596,363	95,600,207	3.845	3	4	Adult August weight (Hap-RHM)	ENSOARG00000020376	TBX15	Highly expressed in sheep muscle (Sheep tissue atlas). Mutations in humans associated with Cousin syndrome (a rare syndrome characterized mainly by short stature at birth, unusual facial appearance and skeletal abnormalities involving the shoulder blades and hips). High expression in fat in humans (HPA RNA-seq normal tissues). Adipose master transcription factor
1	2542	106,366,046	106,386,067	20.022	3	10	Adult August weight (Hap-RHM, SNHap-RHM vs null, SNHap-RHM vs SNP-RHM)			
1	3115	139,277,588	139,295,840	18.253	8	17	Adult August weight (Hap-RHM)			
1	3587	160,031,383	160,095,549	64.167	7	8	Adult August weight (Hap-RHM)			
1	3749	171,350,897	171,455,010	104.114	16	21	Adult August weight (Hap-RHM)	ENSOARG00000018897	IFT57	
1	3843	178,080,084	178,183,944	103.861	15	27	Adult jaw length (Hap-RHM, SNHap-RHM vs null, SNHap-RHM vs SNP-RHM)			
1	3999	187,682,691	187,731,235	48.545	13	14	Adult August weight (Hap-RHM, SNHap-RHM vs null, SNHap-RHM vs SNP-RHM)	ENSOARG00000020233	HEG1	
1	5151	262,392,923	262,395,691	2.769	3	5	Adult August weight (Hap-RHM)	ENSOARG00000011819	AIRE	
1	5152	262,407,194	262,416,861	9.668	6	11	Adult August weight (Hap-RHM, SNHap-RHM vs null, SNHap-RHM vs SNP-RHM)	ENSOARG00000011864	PFKL	
1	5171	263,369,987	263,391,361	21.375	8	26	Adult August weight (Hap-RHM, SNHap-RHM vs null, SNHap-RHM vs SNP-RHM)	ENSOARG00000012584	COL18A1	
1	5173	263,421,836	263,469,212	47.377	10	31	Adult August weight (Hap-RHM, SNHap-RHM vs SNP-RHM)	ENSOARG00000012584	COL18A1	
								ENSOARG00000012653	SLC19A1	
2	294	13,861,125	13,900,430	39.306	14	23	Adult August weight (Hap-RHM, SNHap-RHM vs SNP-RHM)	ENSOARG00000006968	PTPN3	
2	298	13,963,629	14,005,045	41.417	6	20	Adult August weight (Hap-RHM, SNHap-RHM vs SNP-RHM)	ENSOARG00000007009	EPB41L4B	
2	847	38,080,434	38,081,124	0.691	2	4	Adult August weight (Hap-RHM)	ENSOARG00000009659	EPHX2	Linked to obesity in humans
2	1231	58,153,317	58,183,581	30.265	10	14	Adult August weight (Hap-RHM, SNHap-RHM vs SNP-RHM)	ENSOARG00000012371	CEP78	
2	1655	86,113,413	86,164,251	50.839	9	19	Adult August weight (Hap-RHM)	ENSOARG00000021943	U6	
2	1661	86,356,070	86,702,015	345.946	55	57	Adult August weight (Hap-RHM, SNHap-RHM vs null, SNHap-RHM vs SNP-RHM)	ENSOARG00000014133	ADAMTSL1	
2	1686	88,287,607	88,366,762	79.156	14	34	Adult August weight (Hap-RHM, SNHap-RHM vs SNP-RHM)	ENSOARG00000014289		
2	2219	136,485,236	136,485,296	0.061	2	4	Adult August weight (Hap-RHM, SNHap-RHM vs null, SNHap-RHM vs SNP-RHM)			
2	2876	164,795,365	165,011,337	215.973	31	27	Adult August weight (Hap-RHM, SNHap-RHM vs SNP-RHM)	ENSOARG00000009898	GTDC1	
2	4160	230,433,387	230,495,451	62.065	6	9	Lamb hindleg length (Hap-RHM)	ENSOARG00000020608	DNER	
								ENSOARG00000022419	U6	
3	270	15,517,976	15,665,753	147.778	30	39	Lamb jaw length (Hap-RHM)	ENSOARG00000019363	(novel gene)	
								ENSOARG00000025958	(novel gene)	
3	366	20,227,521	20,376,157	148.637	36	45	Adult August weight (Hap-RHM)	ENSOARG00000015997	E2F6	
								ENSOARG00000022085	5s rRNA	
3	1860	105,545,550	105,562,429	16.88	4	5	Adult August weight (Hap-RHM)			
3	2003	120,620,484	121,115,743	495.26	78	73	Adult hindleg length (Hap-RHM, SNHap-RHM vs null, SNHap-RHM vs SNP-RHM)	ENSOARG00000015322	SLC6A15	
								ENSOARG00000015341	TSPAN19	
								ENSOARG00000023767	U6	
3	2046	125,238,635	125,340,391	101.757	18	42	Adult jaw length (Hap-RHM, SNHap-RHM vs null, SNHap-RHM vs SNP-RHM)	ENSOARG00000015607	(novel gene)	
								ENSOARG00000015617	(novel gene)	
3	2217	134,593,364	134,952,658	359.295	46	38	Adult August weight (Hap-RHM, SNHap-RHM vs null, SNHap-RHM vs SNP-RHM)	ENSOARG00000017291	SLC4A8	
								ENSOARG00000017314	GALNT6	
								ENSOARG00000017327	CELA1	
								ENSOARG00000017344	BIN2	
								ENSOARG00000017355	SMAGP	
								ENSOARG00000017368	DAZAP2	
								ENSOARG00000017381	POU6F1	
								ENSOARG00000017390	TFCP2	
								ENSOARG00000017411	CSRNP2	
								ENSOARG00000017425	LETMD1	
3	2749	163,548,241	163,642,434	94.194	22	24	Adult hindleg length (Hap-RHM, SNHap-RHM vs SNP-RHM)	ENSOARG00000018012	LOC101105903	
								ENSOARG00000018031	LOC101107184	
3	2751	163,752,532	163,836,906	84.375	12	10	Adult hindleg length (Hap-RHM, SNHap-RHM vs null, SNHap-RHM vs SNP-RHM)	ENSOARG00000011516	(novel gene)	
3	3432	199,253,201	199,277,862	24.662	8	14	Lamb hindleg length (Hap-RHM)	ENSOARG00000020651	PTPRO	
3	4030	222,455,230	222,490,044	34.815	9	26	Adult August weight (Hap-RHM, SNHap-RHM vs SNP-RHM)			
3	4032	222,503,713	222,513,147	9.435	4	8	Adult August weight (Hap-RHM)			
4	268	13,003,091	13,088,097	85.007	12	24	Adult August weight (Hap-RHM, SNHap-RHM vs SNP-RHM)	ENSOARG00000003766	DYNC1I1	
4	345	16,637,915	16,645,702	7.788	3	6	Adult August weight (Hap-RHM)			
4	1770	97,010,982	97,103,764	92.783	21	32	Adult hindleg length (Hap-RHM, SNHap-RHM vs null, SNHap-RHM vs SNP-RHM)	ENSOARG00000006769	(novel gene)	
6	213	12,129,893	12,195,170	65.278	12	21	Adult August weight (Hap-RHM, SNHap-RHM vs null, SNHap-RHM vs SNP-RHM)	ENSOARG00000018416	CAMK2D	
6	405	19,545,275	19,571,212	25.938	6	11	Adult August weight (Hap-RHM, SNHap-RHM vs null, SNHap-RHM vs SNP-RHM)	ENSOARG00000009956	GSTCD	

6	676	40,594,243	40,598,722	4.48	4	9	Adult August weight (Hap-RHM, SNHap-RHM vs null, SNHap-RHM vs SNP-RHM)			
6	677	40,627,521	40,814,530	187.01	31	29	Adult hindleg length (Hap-RHM, SNHap-RHM vs SNP-RHM)			
6	810	52,709,410	52,878,546	169.137	23	40	Adult August weight (Hap-RHM)			
6	841	54,756,472	54,767,273	10.802	3	6	Adult August weight (Hap-RHM, SNHap-RHM vs null, SNHap-RHM vs SNP-RHM)			
6	855	55,360,902	55,466,024	105.123	16	41	Adult August weight (Hap-RHM, SNHap-RHM vs SNP-RHM)	ENSOARG00000009322	ARAP2	
6	1064	67,126,647	67,275,637	148.991	29	63	Adult foreleg length (Hap-RHM, SNHap-RHM vs null, SNHap-RHM vs SNP-RHM)	ENSOARG00000017345	SLC10A4	
								ENSOARG00000017393	(novel gene)	
								ENSOARG00000017797	FRYL	
6	1652	103,234,182	103,331,382	97.201	30	24	Adult hindleg length (Hap-RHM)	ENSOARG00000007030	EVC2	Humans: his gene encodes a protein that functions in bone formation and skeletal development. Mutations in this gene, as well as in a neighboring gene that lies in a head-to-head configuration, cause Ellis-van Creveld syndrome
7	1	24,558	209,889	185.332	19	142	Adult August weight (Hap-RHM, SNHap-RHM vs null, SNHap-RHM vs SNP-RHM)			
7	161	7,380,870	7,489,218	108.349	26	56	Adult hindleg length (Hap-RHM, SNHap-RHM vs null, SNHap-RHM vs SNP-RHM)	ENSOARG00000016888	IQGAP2	
7	179	8,344,719	8,375,455	30.737	6	22	Adult August weight (Hap-RHM, SNHap-RHM vs null, SNHap-RHM vs SNP-RHM)	ENSOARG00000017022	PDE8B	
								ENSOARG00000026687	(novel gene)	
8	1689	78,683,108	78,734,898	51.791	9	9	Adult August weight (Hap-RHM, SNHap-RHM vs SNP-RHM)			
9	1381	57,985,117	58,057,584	72.468	12	16	Adult August weight (Hap-RHM, SNHap-RHM vs SNP-RHM)	ENSOARG00000009590	COLEC10	
9	1704	72,404,044	72,420,911	16.868	5	7	Adult August weight (Hap-RHM)	ENSOARG00000026544	(novel gene)	
								ENSOARG00000026545	(novel gene)	
9	1723	73,438,286	73,446,206	7.921	4	7	Adult August weight (Hap-RHM)	ENSOARG00000015979	RIMS2	
9	1898	82,835,845	82,875,993	40.149	11	19	Adult August weight (SNHap-RHM vs null)			
9	2032	89,458,815	89,567,544	108.73	24	61	Adult hindleg length (Hap-RHM, SNHap-RHM vs null, SNHap-RHM vs SNP-RHM)			
10	483	18,080,744	18,096,127	15.384	5	11	Adult August weight (Hap-RHM, SNHap-RHM vs null, SNHap-RHM vs SNP-RHM)			
10	489	18,264,001	18,265,718	1.718	2	4	Adult August weight (Hap-RHM, SNHap-RHM vs null, SNHap-RHM vs SNP-RHM)			
10	491	18,306,871	18,342,967	36.097	7	16	Adult August weight (Hap-RHM)			
10	492	18,354,172	18,357,858	3.687	3	6	Adult August weight (Hap-RHM)			
10	790	29,520,015	29,535,013	14.999	3	6	Adult August weight (Hap-RHM, SNHap-RHM vs SNP-RHM)			
10	1194	50,858,554	50,876,668	18.115	7	20	Adult August weight (Hap-RHM)	ENSOARG00000015582	TBC1D4	
10	1587	63,549,495	63,585,886	36.392	6	9	Adult August weight (Hap-RHM)			
10	1628	65,020,171	65,157,788	137.618	14	14	Adult August weight (Hap-RHM)			
10	1629	65,167,961	65,227,127	59.167	10	16	Adult August weight (Hap-RHM, SNHap-RHM vs SNP-RHM)			
11	113	7,422,889	7,457,516	34.628	8	36	Adult foreleg length (Hap-RHM, SNHap-RHM vs null, SNHap-RHM vs SNP-RHM), Adult hindleg length (Hap-RHM, SNHap-RHM vs null, SNHap-RHM vs SNP-RHM)			
11	170	10,398,620	10,447,503	48.884	6	9	Adult August weight (Hap-RHM, SNHap-RHM vs null, SNHap-RHM vs SNP-RHM)	ENSOARG00000014401	RPS6KB1	
								ENSOARG00000014672	RNFT1	
								ENSOARG00000023319	(novel gene)	
11	726	32,805,104	32,988,652	183.549	32	30	Lamb foreleg length (Hap-RHM)	ENSOARG00000004399	UBB	
								ENSOARG00000017009	(novel gene)	
								ENSOARG00000017046	PIGL	
								ENSOARG00000017147	NCOR1	
11	1027	50,247,507	50,268,735	21.229	7	9	Adult jaw length (Hap-RHM)			
12	23	1,315,546	1,498,521	182.976	23	106	Adult foreleg length (Hap-RHM, SNHap-RHM vs null, SNHap-RHM vs SNP-RHM), Adult hindleg length (Hap-RHM, SNHap-RHM vs null, SNHap-RHM vs SNP-RHM)	ENSOARG00000002524	PPP1R15B	PPP1R15B: Human: A Missense Mutation in PPP1R15B Causes a Syndrome Including Diabetes, Short Stature, and Microcephaly
								ENSOARG00000002556	(novel gene)	
								ENSOARG00000002639	PIK3C2B	
12	696	31,886,985	32,003,794	116.81	19	23	Adult August weight (Hap-RHM, SNHap-RHM vs SNP-RHM)	ENSOARG00000006316	SDCCAG8	Sdccag8: humans: linked to obesity
								ENSOARG00000021882	(novel gene)	
12	737	35,365,159	35,408,696	43.538	13	70	Adult August weight (Hap-RHM, SNHap-RHM vs null, SNHap-RHM vs SNP-RHM)	ENSOARG00000010135	SLC19A2	
								ENSOARG00000010726	SELP	
12	1399	68,397,546	68,481,603	84.058	18	9	Adult hindleg length (Hap-RHM)	ENSOARG00000009437	RPS6KC1	
13	208	8,224,592	8,239,443	14.852	2	4	Adult August weight (Hap-RHM, SNHap-RHM vs null, SNHap-RHM vs SNP-RHM)			
13	212	8,321,481	8,340,545	19.065	4	6	Adult August weight (Hap-RHM, SNHap-RHM vs null, SNHap-RHM vs SNP-RHM)			
13	350	14,324,927	14,540,728	215.802	34	46	Adult August weight (Hap-RHM, SNHap-RHM vs null, SNHap-RHM vs SNP-RHM)			
13	488	21,753,962	21,782,926	28.965	4	6	Adult August weight (Hap-RHM, SNHap-RHM vs SNP-RHM)	ENSOARG00000000512	(novel gene)	
13	1025	52,585,011	52,594,105	9.095	3	6	Lamb jaw length (Hap-RHM, SNHap-RHM vs SNP-RHM)			

13	1041	53,300,575	53,763,103	462.529	54	49	Lamb jaw length (Hap-RHM, SNHap-RHM vs SNP-RHM)	ENSOARG00000009948	ZBTB46	LOC101112800/Arf1gap: Mouse:Located in postsynaptic density. Is expressed in axial skeleton;
								ENSOARG00000010128	ZGPAT	craniocervical region bone; inner ear; and lower jaw mesenchyme
								ENSOARG00000010188	ARFRP1	
								ENSOARG00000010322	TNFRSF6B	
								ENSOARG00000010368	RTEL1	
								ENSOARG00000010555	STMN3	
								ENSOARG00000010596	GMEB2	
								ENSOARG00000010686	FNDC11	
								ENSOARG00000010780	SRMS	
								ENSOARG00000010950	PTK6	
								ENSOARG00000010995	(novel gene)	
								ENSOARG00000011052	EEF1A2	
								ENSOARG00000011107	KCNQ2	
								ENSOARG00000011195	CHRNA4	
								ENSOARG00000011265	(novel gene)	
								ENSOARG00000011406	LOC101112800	
								ENSOARG00000011497	(novel gene)	
								ENSOARG00000011555	(novel gene)	
								ENSOARG00000011588	BIRC7	
								ENSOARG00000011639	YTHDF1	
								ENSOARG00000011782	SLC17A9	
								ENSOARG00000022609	(novel gene)	
								ENSOARG00000026240	(novel gene)	
14	666	29,320,129	29,404,807	84.679	13	56	Adult August weight (Hap-RHM, SNHap-RHM vs null, SNHap-RHM vs SNP-RHM)			
14	762	34,218,884	34,718,824	499.941	59	19	Lamb jaw length (Hap-RHM)	ENSOARG00000002860	FHOD1	
								ENSOARG00000002877	SLC9A5	
								ENSOARG00000002900	PLEKHG4	
								ENSOARG00000002928	KCTD19	
								ENSOARG00000002953	LRRC36	
								ENSOARG00000002965	TPPP3	
								ENSOARG00000002972	ZDHHC1	
								ENSOARG00000002986	HSD11B2	
								ENSOARG00000002996	ATP6V0D1	
								ENSOARG00000003006	AGRP	
								ENSOARG00000003027	RIPOR1	
								ENSOARG00000003053	CTCF	
								ENSOARG00000003079	CARMIL2	
								ENSOARG00000003098	ACD	
								ENSOARG00000003109	PARD6A	
								ENSOARG00000003118	ENKD1	
								ENSOARG00000003128	C14H16orf86	
								ENSOARG00000003135	GFOD2	
								ENSOARG00000003145	RANBP10	
								ENSOARG00000003171	TSNAXIP1	
								ENSOARG00000003186	CENPT	
								ENSOARG00000003196	(novel gene)	
								ENSOARG00000003200	NUTF2	
								ENSOARG00000003220	EDC4	
15	256	16,293,816	16,793,796	499.981	44	37	Adult hindleg length (Hap-RHM, SNHap-RHM vs null, SNHap-RHM vs SNP-RHM)	ENSOARG00000010464	ALKBH8	
								ENSOARG00000010653	ELMOD1	
								ENSOARG00000016955	SLN	
								ENSOARG00000023352	(novel gene)	
15	1109	61,307,131	61,476,746	169.616	23	38	Adult August weight (Hap-RHM, SNHap-RHM vs null, SNHap-RHM vs SNP-RHM)	ENSOARG00000016334	WT1	
								ENSOARG00000021466	(novel gene)	
								ENSOARG00000021578	(novel gene)	
								ENSOARG00000022621	(novel gene)	
								ENSOARG00000023601	(novel gene)	
								ENSOARG00000024560	(novel gene)	
								ENSOARG00000024644	(novel gene)	
16	1326	68,447,430	68,454,153	6.724	3	4	Lamb metacarpal length (SNP-RHM)	ENSOARG00000015559	(novel gene)	
16	1333	68,687,286	68,705,421	18.136	5	8	Adult metacarpal length (SNP-RHM)			
16	1340	68,915,832	68,981,502	65.671	13	27	Lamb metacarpal length (SNP-RHM, Hap-RHM, SNHap-RHM vs null),			
							Adult metacarpal length (SNP-RHM, Hap-RHM, SNHap-RHM vs null)			
16	1346	69,129,929	69,138,953	9.025	6	12	Lamb metacarpal length (SNP-RHM, Hap-RHM),			
							Adult metacarpal length (SNP-RHM, Hap-RHM, SNHap-RHM vs null)			
16	1347	69,151,199	69,158,314	7.116	3	5	Lamb metacarpal length (SNP-RHM, Hap-RHM)			
16	1348	69,160,398	69,181,657	21.26	6	11	Lamb metacarpal length (SNP-RHM, Hap-RHM),			
							Adult metacarpal length (SNP-RHM, Hap-RHM, SNHap-RHM vs null)			
16	1352	69,226,093	69,306,236	80.144	18	29	Lamb metacarpal length (SNP-RHM, Hap-RHM, SNHap-RHM vs null),	ENSOARG00000005781	(novel gene)	
							Adult metacarpal length (SNP-RHM, Hap-RHM, SNHap-RHM vs null)	ENSOARG00000026985	(novel gene)	

16	1360	69,425,086	69,574,670	149.585	30	32	Lamb metacarpal length (SNP-RHM, Hap-RHM, SNHap-RHM vs null),			
							Adult metacarpal length (SNP-RHM, Hap-RHM, SNHap-RHM vs null)			
16	1363	69,667,639	69,754,149	86.511	17	18	Lamb metacarpal length (SNP-RHM, Hap-RHM, SNHap-RHM vs null),			
							Adult metacarpal length (SNP-RHM, Hap-RHM),			
							Adult hindleg length (SNP-RHM)			
16	1367	69,839,345	69,853,299	13.955	6	9	Lamb metacarpal length (SNP-RHM, Hap-RHM, SNHap-RHM vs null),			
							Adult metacarpal length (SNP-RHM, Hap-RHM, SNHap-RHM vs null),			
							Adult hindleg length (SNP-RHM, Hap-RHM, SNHap-RHM vs null)			
16	1368	69,856,342	69,943,027	86.686	17	23	Lamb metacarpal length (SNP-RHM, Hap-RHM),			
							Adult metacarpal length (SNP-RHM, Hap-RHM, SNHap-RHM vs null)			
16	1379	70,307,056	70,368,449	61.394	15	17	Lamb metacarpal length (SNP-RHM, Hap-RHM, SNHap-RHM vs null),			
							Adult metacarpal length (SNP-RHM, Hap-RHM, SNHap-RHM vs null)			
16	1382	70,412,884	70,442,751	29.868	6	16	Lamb metacarpal length (SNP-RHM, Hap-RHM, SNHap-RHM vs null),			
							Adult metacarpal length (SNP-RHM, Hap-RHM, SNHap-RHM vs null),			
							Adult hindleg length (SNP-RHM)			
16	1384	70,469,116	70,480,771	11.656	5	6	Adult metacarpal length (SNP-RHM, Hap-RHM, SNHap-RHM vs null)	ENSOARG00000015565	(novel gene)	
								ENSOARG00000015599	CCDC127	
								ENSOARG00000026989	(novel gene)	
16	1385	70,492,788	70,861,290	368.503	32	62	Lamb metacarpal length (SNP-RHM, Hap-RHM, SNHap-RHM vs null),	ENSOARG00000015619	(novel gene)	
							Adult metacarpal length (SNP-RHM, SNHap-RHM vs null)	ENSOARG00000015678	SDHA	
								ENSOARG00000015729	PDCD6	
								ENSOARG00000015756	(novel gene)	
								ENSOARG00000015791	(novel gene)	
								ENSOARG00000015862	EXOC3	
								ENSOARG00000015903	SLC9A3	
								ENSOARG00000015928	CEP72	
								ENSOARG00000015975	TPPP	
								ENSOARG00000021220	BRD9	
								ENSOARG00000026989	5S rRNA	
								ENSOARG00000026990	(novel gene)	
								ENSOARG00000026991	(novel gene)	
								ENSOARG00000026992	(novel gene)	
16	1390	71,032,937	71,037,268	4.332	6	7	Lamb metacarpal length (SNP-RHM, Hap-RHM, SNHap-RHM vs null),	ENSOARG00000016124	(novel gene)	
							Adult hindleg length (SNP-RHM, Hap-RHM)			
16	1393	71,117,303	71,134,260	16.958	4	9	Lamb metacarpal length (SNP-RHM, Hap-RHM, SNHap-RHM vs null)			
16	1394	71,135,107	71,417,145	282.039	24	39	Lamb metacarpal length (SNP-RHM),	ENSOARG00000016135	(novel gene)	
							Adult metacarpal length (SNP-RHM, SNHap-RHM vs null)	ENSOARG00000016170	IRX4	
								ENSOARG00000021595	5S rRNA	
								ENSOARG00000026994	(novel gene)	
								ENSOARG00000026995	(novel gene)	
								ENSOARG00000026996	(novel gene)	
								ENSOARG00000026997	(novel gene)	
								ENSOARG00000026998	(novel gene)	
								ENSOARG00000026999	(novel gene)	
16	1398	71,538,789	71,603,934	65.146	11	10	Adult metacarpal length (SNP-RHM, Hap-RHM)	ENSOARG00000016211	SLC6A3	
								ENSOARG00000027001	(novel gene)	
17	570	17,198,504	17,283,637	85.134	12	25	Adult hindleg length (Hap-RHM)	ENSOARG00000012885	(novel gene)	
17	597	18,436,762	18,537,277	100.516	14	49	Adult August weight (Hap-RHM, SNHap-RHM vs SNP-RHM)	ENSOARG00000025659	(novel gene)	
17	923	39,422,031	39,459,777	37.747	9	13	Lamb jaw length (Hap-RHM)	ENSOARG00000002302	RAPGEF2	
17	1368	57,691,208	57,747,777	56.57	11	15	Adult hindleg length (Hap-RHM, SNHap-RHM vs null, SNHap-RHM vs SNP-RHM)	ENSOARG00000005170	RNFT2	
17	1369	57,758,713	57,776,113	17.401	5	5	Adult hindleg length (Hap-RHM)	ENSOARG00000004678	(novel gene)	
17	1669	71,033,562	71,045,469	11.908	2	4	Adult August weight (Hap-RHM, SNHap-RHM vs null, SNHap-RHM vs SNP-RHM)	ENSOARG00000014555	(novel gene)	
								ENSOARG00000025708	(novel gene)	
17	1672	71,176,215	71,176,575	0.361	2	4	Adult August weight (Hap-RHM)	ENSOARG00000015034	(novel gene)	
18	665	36,416,557	36,442,672	26.116	6	13	Adult jaw length (Hap-RHM)			
18	1248	62,650,233	62,714,362	64.13	14	41	Adult hindleg length (Hap-RHM, SNHap-RHM vs null, SNHap-RHM vs SNP-RHM)			
19	700	37,999,202	38,018,884	19.683	6	8	Adult August weight (Hap-RHM, SNHap-RHM vs null, SNHap-RHM vs SNP-RHM)	ENSOARG00000011630	SYNPR	
19	915	48,504,989	48,559,115	54.127	12	19	Lamb metacarpal length (SNP-RHM)	ENSOARG00000005161	DNAH1	
19	921	48,699,277	48,839,372	140.096	19	22	Lamb metacarpal length (SNP-RHM, SNHap-RHM vs null)	ENSOARG00000006352	POC1A	POC1A: Mutations in this gene result in short stature, onychodysplasia, facial dysmorphism, and
								ENSOARG00000006432	DUSP7	hypotrichosis (SOFT) syndrome. (Mouse) Acts upstream of or within several processes, including
								ENSOARG00000026657	(novel gene)	growth plate cartilage chondrocyte development
19	923	48,882,534	48,959,233	76.7	22	32	Lamb metacarpal length (SNP-RHM, SNHap-RHM vs null)	ENSOARG00000006456	RPL29	
								ENSOARG00000006479	ABHD14A	
								ENSOARG00000006536	ABHD14B	
								ENSOARG00000006603	PCBP4	
								ENSOARG00000006608	(novel gene)	
								ENSOARG00000006615	(novel gene)	
								ENSOARG00000006651	(novel gene)	
								ENSOARG00000006698	RRP9	

19	924	48,961,593	49,289,143	327.551	45	83	Lamb metacarpal length (SNHap-RHM vs null, SNHap-RHM vs Hap-RHM)	ENSOARG00000006698	RRP9	
								ENSOARG00000006825	IQCF1	
								ENSOARG00000006874	LOC101115831	
								ENSOARG00000006884	IQCF2	
								ENSOARG00000006886	IQCF3	
								ENSOARG00000006909	(novel gene)	
								ENSOARG00000006915	IQCF6	
								ENSOARG00000006974	GRM2	
								ENSOARG00000007079	TEX264	
								ENSOARG00000007264	RAD54L2	
								ENSOARG00000007546	DCAF1	
								ENSOARG00000014150	LOC101117539	
								ENSOARG00000023520	U4	
19	926	49,334,367	49,391,499	57.133	7	10	Lamb metacarpal length (SNP-RHM, Hap-RHM, SNHap-RHM vs null)	ENSOARG00000007757	RBM15B	
								ENSOARG00000007779	MANF	
								ENSOARG00000008062	DOCK3	
19	931	49,807,150	49,823,282	16.133	6	10	Lamb metacarpal length (SNP-RHM)			
19	932	49,834,431	49,918,951	84.521	13	15	Lamb metacarpal length (SNP-RHM, Hap-RHM, SNHap-RHM vs null)	ENSOARG00000009120	CACNA2D2	
19	935	50,126,233	50,427,222	300.99	41	27	Lamb metacarpal length (SNP-RHM, Hap-RHM, SNHap-RHM vs null)	ENSOARG00000010649	SEMA3F	
								ENSOARG00000010923	RBM5	
								ENSOARG00000011125	RBM6	
								ENSOARG00000011281	MON1A	
								ENSOARG00000011352	MST1R	
								ENSOARG00000011510	CAMKV	
								ENSOARG00000011570	TRAIP	
								ENSOARG00000011997	UBA7	
								ENSOARG00000012365	INKA1	
								ENSOARG00000012413	IP6K1	
								ENSOARG00000012451	GMPPB	
								ENSOARG00000022525	U6	
19	937	50,455,405	50,545,393	89.989	12	53	Lamb metacarpal length (SNP-RHM, Hap-RHM, SNHap-RHM vs null)	ENSOARG00000013151	MST1	
								ENSOARG00000013387	APEH	
								ENSOARG00000013564	BSN	
19	938	50,546,414	50,574,275	27.862	8	16	Lamb metacarpal length (SNP-RHM, Hap-RHM, SNHap-RHM vs null)	ENSOARG00000013645	DAG1	
19	940	50,594,654	50,934,237	339.584	50	25	Lamb metacarpal length (SNP-RHM, Hap-RHM, SNHap-RHM vs null)	ENSOARG00000013728	NICN1	TCTA + RHOA : Humans + mice: Involved in negative regulation of osteoclast differentiation and osteoclast fusion
								ENSOARG00000013773	AMT	
								ENSOARG00000013798	TCTA	
								ENSOARG00000013860	RHOA	
								ENSOARG00000013960	USP4	
								ENSOARG00000014215	C19H3orf62	
								ENSOARG00000014273	IHO1	
								ENSOARG00000014296	KLHDC8B	
								ENSOARG00000014388	CCDC71	
								ENSOARG00000014559	LOC101106719	
								ENSOARG00000014927	USP19	
								ENSOARG00000015195	QARS1	
								ENSOARG00000015363	QRICH1	
								ENSOARG00000015447	IMPDH2	
								ENSOARG00000015657	NDUFAF3	
								ENSOARG00000015672	DALRD3	
								ENSOARG00000015805	WDR6	
								ENSOARG00000015842	P4HTM	
								ENSOARG00000022437	(novel gene)	
								ENSOARG00000023735	Oar-mir-191	
								ENSOARG00000023885	U6	
19	941	50,982,456	51,125,248	142.793	23	30	Lamb metacarpal length (SNP-RHM, SNHap-RHM vs null)	ENSOARG00000016084	SLC25A20	
								ENSOARG00000016132	PRKAR2A	
19	942	51,143,353	51,144,906	1.554	4	4	Lamb metacarpal length (SNP-RHM, Hap-RHM)	ENSOARG00000016244	NCKIPSD	
19	943	51,154,170	51,171,583	17.414	3	5	Lamb metacarpal length (SNP-RHM, Hap-RHM, SNHap-RHM vs null)	ENSOARG00000016244	NCKIPSD	
								ENSOARG00000016424	CELSR3	

19	945	51,192,408	51,582,246	389.839	52	60	Lamb metacarpal length (SNHap-RHM vs null, SNHap-RHM vs Hap-RHM)	ENSOARG00000000092	CELSR3	PLXNB1: Mouse: Involved in several processes, including negative regulation of osteoblast proliferation
								ENSOARG00000000153	SHISA5	
								ENSOARG00000000255	TREX1	
								ENSOARG00000000409	ATRIP	
								ENSOARG00000000605	CCDC51	
								ENSOARG00000000999	PLXNB1	
								ENSOARG00000001093	SPINK8	
								ENSOARG00000001285	NME6	
								ENSOARG00000001392	LOC101112427	
								ENSOARG00000001481	(novel gene)	
								ENSOARG00000001605	(novel gene)	
								ENSOARG00000001802	LOC105607776	
								ENSOARG00000001969	CATHL3	
								ENSOARG00000002027	BAC5	
								ENSOARG00000014171	(novel gene)	
								ENSOARG00000016424	UCN2	
								ENSOARG00000016539	SLC26A6	
								ENSOARG00000016645	TMEM89	
								ENSOARG00000016718	LOC101110095	
								ENSOARG00000017257	COL7A1	
								ENSOARG00000017897	PFKFB4	
								ENSOARG00000021670	(novel gene)	
								ENSOARG00000026659	(novel gene)	
								ENSOARG00000026660	(novel gene)	
								ENSOARG00000026661	(novel gene)	
19	946	51,583,587	51,641,462	57.876	14	22	Lamb metacarpal length (SNP-RHM, Hap-RHM, SNHap-RHM vs null)	ENSOARG00000002154	SC5	
								ENSOARG00000002373	CDC25A	
								ENSOARG00000022608	5S rRNA	
19	948	51,665,021	51,881,263	216.243	40	64	Lamb metacarpal length (SNP-RHM, SNHap-RHM vs null), Adult metacarpal length (SNP-RHM, SNHap-RHM vs null)	ENSOARG00000002647	MAP4	
								ENSOARG00000002814	(novel gene)	
								ENSOARG00000002897	DHX30	
								ENSOARG00000024996	(novel gene)	
19	950	51,908,871	52,340,442	431.572	66	72	Lamb metacarpal length (SNP-RHM, SNHap-RHM vs null, SNHap-RHM vs Hap-RHM), Adult metacarpal length (SNP-RHM, SNHap-RHM vs null)	ENSOARG00000003267	SMARCC1	
								ENSOARG00000003512	CSPG5	
								ENSOARG00000003560	ELP6	
								ENSOARG00000003881	SCAP	
								ENSOARG00000004081	(novel gene)	
								ENSOARG00000004151	LOC101122819	
								ENSOARG00000004308	KLHL18	
								ENSOARG00000004598	KIF9	
								ENSOARG00000024491	U6	
								ENSOARG00000026662	(novel gene)	
19	952	52,376,602	52,543,759	167.158	37	52	Lamb metacarpal length (SNP-RHM, SNHap-RHM vs null, SNHap-RHM vs Hap-RHM), Adult metacarpal length (SNP-RHM, SNHap-RHM vs null)	ENSOARG00000005054	SETD2	PTH1R: Humans: Defects in this receptor are known to be the cause of Jansen's metaphyseal chondrodysplasia (JMC), chondrodysplasia Blomstrand type (BOCD), as well as enchodromatosis. In bone, it is expressed on the surface of osteoblasts. When the receptor is activated through PTH binding, osteoblasts express RANKL (Receptor Activator of Nuclear Factor kB Ligand), which binds to RANK (Receptor Activator of Nuclear Factor kB) on osteoclasts. This turns on osteoclasts to ultimately increase the resorption rate.
								ENSOARG00000005435	LOC101114909	
								ENSOARG00000005857	NBEAL2	
								ENSOARG00000006426	CCDC12	
								ENSOARG00000006638	PTH1R	
19	954	52,570,585	52,595,896	25.312	7	10	Lamb metacarpal length (SNP-RHM, SNHap-RHM vs null)	ENSOARG00000007025	LOC101115659	
19	957	52,659,885	52,732,395	72.511	20	28	Lamb metacarpal length (SNP-RHM, Hap-RHM, SNHap-RHM vs null)	ENSOARG00000007290	LOC101102765	
								ENSOARG00000007381	PRSS50	
								ENSOARG00000007470	TMIE	
								ENSOARG00000007704	(novel gene)	
								ENSOARG00000014187	(novel gene)	
								ENSOARG00000026663	ALS2CL	
19	958	52,733,279	52,798,829	65.551	15	20	Lamb metacarpal length (SNP-RHM, Hap-RHM, SNHap-RHM vs null), Adult metacarpal length (SNP-RHM, SNHap-RHM vs null)	ENSOARG00000007704	ALS2CL	
								ENSOARG00000008087	FAM240A	
								ENSOARG00000008117	CRIPTO	
19	964	52,912,220	53,099,344	187.125	37	45	Lamb metacarpal length (SNP-RHM, SNHap-RHM vs null)	ENSOARG00000014203	CCRL2	
								ENSOARG00000014214	CCR5	
								ENSOARG00000014236	LOC101117706	
								ENSOARG00000014244	CCR3	
19	969	53,372,619	53,548,690	176.072	29	52	Lamb metacarpal length (SNP-RHM)	ENSOARG00000009571	LZTFL1	LIMD1: Mouse: Involved in negative regulation of canonical Wnt signaling pathway; negative regulation of osteoblast differentiation; and osteoblast development. Expressed in limb
								ENSOARG00000009813	SLC6A20	
								ENSOARG00000010162	SACM1L	
								ENSOARG00000010390	LIMD1	
20	3	358,178	802,423	444.246	42	143	Adult hindleg length (Hap-RHM, SNHap-RHM vs null, SNHap-RHM vs SNP-RHM)	ENSOARG00000005603	KHDRBS2	
								ENSOARG00000024249	U6	
20	8	1,254,840	1,315,189	60.35	13	13	Adult hindleg length (Hap-RHM, SNHap-RHM vs null, SNHap-RHM vs SNP-RHM)			
20	263	14,097,841	14,118,776	20.936	6	8	Adult August weight (Hap-RHM, SNHap-RHM vs SNP-RHM)			

20	273	14,428,550	14,485,702	57.153	10	16	Adult August weight (Hap-RHM)		
20	1059	47,667,243	47,726,425	59.183	10	22	Adult hindleg length (Hap-RHM)		
20	1075	48,121,607	48,242,296	120.69	24	63	Adult hindleg length (Hap-RHM, SNHap-RHM vs SNP-RHM)	ENSOARG00000018718	(novel gene)
21	307	13,200,687	13,254,516	53.83	8	10	Adult hindleg length (Hap-RHM)		
21	746	45,263,507	45,345,862	82.356	23	19	Adult August weight (Hap-RHM)	ENSOARG00000015793	PPP6R3
22	138	8,349,025	8,370,713	21.689	5	12	Adult August weight (Hap-RHM, SNHap-RHM vs null, SNHap-RHM vs SNP-RHM)		
22	140	8,379,465	8,400,165	20.701	5	11	Adult August weight (Hap-RHM, SNHap-RHM vs SNP-RHM)	ENSOARG00000013806	ASAH2
22	327	13,291,916	13,300,911	8.996	3	6	Adult August weight (Hap-RHM)	ENSOARG00000016671	CPEB3
22	1167	44,452,770	44,548,081	95.312	21	52	Adult August weight (Hap-RHM)	ENSOARG00000012241	ADAM12
22	1246	50,486,318	50,820,764	334.447	43	62	Adult August weight (Hap-RHM, SNHap-RHM vs SNP-RHM)	ENSOARG00000000042	CYP2E1
								ENSOARG00000000369	ECHS1
								ENSOARG00000000663	FUOM
								ENSOARG00000000727	(novel gene)
								ENSOARG00000000817	CALY
								ENSOARG00000000965	ZNF511
								ENSOARG00000001302	TUBGCP2
								ENSOARG00000014956	(novel gene)
								ENSOARG00000014969	(novel gene)
								ENSOARG00000014978	(novel gene)
								ENSOARG00000017206	CFAP46
								ENSOARG00000018365	(novel gene)
								ENSOARG00000018459	MTG1
								ENSOARG00000018583	(novel gene)
								ENSOARG00000018620	(novel gene)
								ENSOARG00000026777	(novel gene)
								ENSOARG00000026779	(novel gene)
23	342	14,087,536	14,348,846	261.311	13	7	Adult jaw length (Hap-RHM)	ENSOARG00000003785	(novel gene)
								ENSOARG00000005184	PIK3C3
23	434	21,076,868	21,139,467	62.6	12	32	Adult jaw length (Hap-RHM, SNHap-RHM vs SNP-RHM)	ENSOARG00000021861	U6
23	642	39,064,510	39,116,473	51.964	10	15	Adult August weight (Hap-RHM, SNHap-RHM vs null, SNHap-RHM vs SNP-RHM)	ENSOARG00000026186	(novel gene)
23	643	39,126,867	39,137,482	10.616	4	5	Adult August weight (Hap-RHM, SNHap-RHM vs null, SNHap-RHM vs SNP-RHM)	ENSOARG00000026186	(novel gene)
23	651	39,387,808	39,419,963	32.156	6	11	Adult August weight (Hap-RHM)		
23	667	39,908,616	39,931,884	23.269	8	13	Adult August weight (Hap-RHM, SNHap-RHM vs null, SNHap-RHM vs SNP-RHM)		
23	673	40,052,504	40,140,303	87.8	16	34	Adult August weight (Hap-RHM, SNHap-RHM vs null, SNHap-RHM vs SNP-RHM)	ENSOARG00000000085	ARHGAP28
								ENSOARG00000026188	(novel gene)
23	1020	58,109,141	58,167,269	58.129	12	61	Adult foreleg length (Hap-RHM, SNHap-RHM vs null, SNHap-RHM vs SNP-RHM)	ENSOARG00000005569	ZNF532
24	489	41,675,204	41,759,916	84.713	9	36	Adult August weight (Hap-RHM, SNHap-RHM vs null, SNHap-RHM vs SNP-RHM)	ENSOARG00000006153	PDGFA
								ENSOARG00000025920	(novel gene)
25	82	4,586,431	4,883,217	296.787	50	50	Adult August weight (Hap-RHM, SNHap-RHM vs SNP-RHM)	ENSOARG00000003291	DISC1
								ENSOARG00000023491	(novel gene)
25	839	45,065,687	45,210,626	144.94	30	319	Adult hindleg length (Hap-RHM, SNHap-RHM vs SNP-RHM)	ENSOARG00000002999	ZNF32
								ENSOARG00000026418	(novel gene)
26	259	13,997,630	14,095,506	97.877	13	31	Adult foreleg length (Hap-RHM, SNHap-RHM vs null, SNHap-RHM vs SNP-RHM)		
26	288	15,931,720	15,994,925	63.206	11	33	Adult hindleg length (Hap-RHM, SNHap-RHM vs null, SNHap-RHM vs SNP-RHM)		

Supplementary Table 1 – Full list of haplotype blocks for which RHM significantly improved model fit for at least one trait. From left to right: chromosome, haplotype block number, haplotype block start (bp), haplotype block end (bp), length of haplotype block (Kb), number of SNPs in the block, number of haplotype alleles in the block, the trait-RHM model combinations that showed significant improvement in model fit, the Ensembl gene IDs of the genes located in or overlapping the block, the NCBI gene names, and any functional data relevant to the traits.

Chromosome	Block start (bp)	Block end (bp)	SNP-RHM	Hap-RHM	SNHap-RHM vs null	SNHap-RHM vs Hap-RHM	SNHap-RHM vs SNP-RHM
1	73,687,439	73,757,464	Not signifiant	7.98E-07	Model failed	Model failed	Model failed
11	32,805,104	32,988,652	Not signifiant	1.45E-07	Model failed	Model failed	Model failed

Supplementary Table 2 – Full list of haplotype blocks for which at least one RHM method significantly improved model fit for lamb foreleg length. From left to right: chromosome, the haplotype block start (bp), haplotype block end (bp), and whether the inclusion of the regional GRM(s) significantly improved model fit (green), did not significantly improve model fit (yellow), or if a model failed to converge (red) for SNP-RHM, Hap-RHM, SNHap-RHM when compared to the null model, SNHap-RHM when compared to Hap-RHM, and SNHap-RHM when compared to SNP-RHM.

Chromosome	Block start (bp)	Block end (bp)	SNP-RHM	Hap-RHM	SNHap-RHM vs null	SNHap-RHM vs Hap-RHM	SNHap-RHM vs SNP-RHM
2	230,433,387	230,495,451	Not significant	4.56E-07	Model failed	Model failed	Model failed
3	199,253,201	199,277,862	Not significant	9.29E-07	Model failed	Model failed	Model failed

Supplementary Table 3 – Full list of haplotype blocks for which at least one RHM method significantly improved model fit for lamb hindleg length. From left to right: chromosome, the haplotype block start (bp), haplotype block end (bp), and whether the inclusion of the regional GRM(s) significantly improved model fit (green), did not significantly improve model fit (yellow), or if a model failed to converge (red) for SNP-RHM, Hap-RHM, SNHap-RHM when compared to the null model, SNHap-RHM when compared to Hap-RHM, and SNHap-RHM when compared to SNP-RHM.

hromosom	Block start (bp)	Block end (bp)	SNP-RHM	Hap-RHM	SNHap-RHM vs null	SNHap-RHM vs Hap-RHM	SNHap-RHM vs SNP-RHM
16	68447430	68454153	8.34E-07	Not significant	Not significant	Not significant	Not significant
16	68915832	68981502	6.16E-10	3.48E-07	3.03E-09	Not significant	Not significant
16	69129929	69138953	1.03E-11	2.86E-09	Model failed	Model failed	Model failed
16	69151199	69158314	3.56E-10	3.45E-11	Model failed	Model failed	Model failed
16	69160398	69181657	9.76E-11	1.64E-10	Model failed	Model failed	Model failed
16	69226093	69306236	9.81E-10	2.16E-07	8.68E-07	Not significant	Not significant
16	69425086	69574670	2.95E-12	3.25E-09	1.12E-11	Not significant	Not significant
**16**	**69667639**	**69754149**	**9.96E-13**	**2.13E-09**	**5.39E-12**	**Not significant**	**Not significant**
16	69839345	69853299	4.07E-13	6.06E-12	2.29E-12	Not significant	Not significant
16	69856342	69943027	2.77E-13	1.58E-09	Model failed	Model failed	Model failed
16	70307056	70368449	7.17E-10	3.98E-08	5.20E-09	Not significant	Not significant
16	70412884	70442751	1.33E-11	6.34E-10	7.01E-11	Not significant	Not significant
16	70492788	70861290	2.44E-11	1.65E-08	5.20E-11	Not significant	Not significant
16	71032937	71037268	6.40E-12	1.69E-10	3.42E-11	Not significant	Not significant
16	71117303	71134260	1.06E-10	1.09E-09	5.17E-10	Not significant	Not significant
16	71135107	71417145	2.25E-08	Not significant	Model failed	Model failed	Model failed
19	48504989	48559115	2.73E-07	Not significant	Not significant	Not significant	Not significant
19	48699277	48839372	1.05E-08	Not significant	4.18E-08	Not significant	Not significant
19	48882534	48959233	1.27E-07	Not significant	5.63E-07	Not significant	Not significant
19	48961593	49289143	3.21E-08	Not significant	1.59E-07	5.77E-07	Not significant
19	49334367	49391499	4.03E-07	5.78E-07	9.51E-07	Not significant	Not significant
19	49807150	49823282	7.63E-07	Not significant	Model failed	Model failed	Model failed
19	49834431	49918951	1.33E-09	6.94E-09	6.39E-09	Not significant	Not significant
19	50126233	50427222	1.82E-08	1.57E-08	3.93E-08	Not significant	Not significant
19	50455405	50545393	1.25E-09	5.38E-07	4.01E-09	Not significant	Not significant
19	50546414	50574275	5.21E-08	7.71E-07	1.56E-07	Not significant	Not significant
19	50594654	50934237	3.86E-10	9.14E-09	1.51E-09	Not significant	Not significant
19	50982456	51125248	8.01E-10	Not significant	3.86E-09	Not significant	Not significant
19	51143353	51144906	6.33E-09	3.19E-09	Model failed	Model failed	Model failed
19	51154170	51171583	3.81E-10	3.39E-10	1.68E-09	Not significant	Not significant
19	51192408	51582246	3.00E-10	Not significant	1.49E-09	9.38E-07	Not significant
19	51583587	51641462	2.16E-09	3.33E-08	1.03E-08	Not significant	Not significant
19	51665021	51881263	2.16E-09	Not significant	9.94E-09	Not significant	Not significant
19	51908871	52340442	7.18E-11	Not significant	3.68E-10	1.27E-07	Not significant
**19**	**52376602**	**52543759**	**3.43E-11**	**Not significant**	**1.78E-10**	**4.53E-07**	**Not significant**
19	52570585	52595896	1.97E-07	Not significant	8.49E-07	Not significant	Not significant
19	52659885	52732395	5.73E-08	5.25E-07	2.54E-07	Not significant	Not significant
19	52733279	52798829	7.13E-11	2.87E-08	3.65E-10	Not significant	Not significant
19	52912220	53099344	1.24E-07	Not significant	5.41E-07	Not significant	Not significant
19	53372619	53548690	3.36E-07	Not significant	Model failed	Model failed	Model failed

Supplementary Table 4 – Full list of haplotype blocks for which at least one RHM method significantly improved model fit for lamb metacarpal length. From left to right: chromosome, the haplotype block start (bp), haplotype block end (bp), and whether the inclusion of the regional GRM(s) significantly improved model fit (green), did not significantly improve model fit (yellow), or if a model failed to converge (red) for SNP-RHM, Hap-RHM, SNHap-RHM when compared to the null model, SNHap-RHM when compared to Hap-RHM, and SNHap-RHM when compared to SNP-RHM. Bold indicates that the block contains a top GWAS SNP for this trait.

Chromosome	Block start (bp)	Block end (bp)	SNP-RHM	Hap-RHM	SNHap-RHM vs null	SNHap-RHM vs Hap-RHM	SNHap-RHM vs SNP-RHM
3	15,517,976	15,665,753	Not significant	4.93E-07	Model failed	Model failed	Model failed
13	52,585,011	52,594,105	Not significant	2.49E-07	Not significant	Not significant	5.20E-07
13	53,300,575	53,763,103	Not significant	5.08E-07	Not significant	Not significant	5.46E-07
14	34,218,884	34,718,824	Not significant	1.33E-08	Model failed	Model failed	Model failed
17	39,422,031	39,459,777	Not significant	1.14E-07	Model failed	Model failed	Model failed

Supplementary Table 5 – Full list of haplotype blocks for which at least one RHM method significantly improved model fit for lamb jaw length. From left to right: chromosome, the haplotype block start (bp), haplotype block end (bp), and whether the inclusion of the regional GRM(s) significantly improved model fit (green), did not significantly improve model fit (yellow), or if a model failed to converge (red) for SNP-RHM, Hap-RHM, SNHap-RHM when compared to the null model, SNHap-RHM when compared to Hap-RHM, and SNHap-RHM when compared to SNP-RHM.

Chromosome	Block start (bp)	Block end (bp)	SNP-RHM	Hap-RHM	SNHap-RHM vs null	SNHap-RHM vs Hap-RHM	SNHap-RHM vs SNP-RHM
1	40,773,086	40,841,455	Not significant	8.75E-08	4.92E-07	Not significant	1.84E-07
1	40,847,219	40,872,161	Not significant	3.67E-07	Model failed	Model failed	Model failed
1	41,046,557	41,094,853	Not significant	2.77E-07	Not significant	Not significant	4.34E-07
1	41,690,586	41,727,381	Not significant	4.50E-08	2.47E-07	Not significant	5.58E-08
1	41,894,503	41,896,031	Not significant	1.74E-08	1.23E-07	Not significant	3.21E-08
1	51,825,023	51,860,711	Not significant	6.22E-07	Not significant	Not significant	Not significant
1	82,068,913	82,193,611	Not significant	8.74E-08	7.93E-07	Not significant	1.84E-07
1	92,789,120	92,822,945	Not significant	5.24E-07	Not significant	Not significant	7.82E-07
1	95,596,363	95,600,207	Model failed	4.44E-07	Model failed	Model failed	Model failed
1	106,366,046	106,386,067	Not significant	9.19E-08	5.64E-07	Not significant	1.30E-07
1	139,277,588	139,295,840	Not significant	9.02E-07	Model failed	Model failed	Model failed
1	160,031,383	160,095,549	Not significant	1.36E-08	Not significant	Not significant	Not significant
1	171,350,897	171,455,010	Not significant	7.97E-07	Not significant	Not significant	Not significant
1	187,682,691	187,731,235	Not significant	1.29E-08	6.25E-08	Not significant	2.39E-07
1	262,392,923	262,395,691	Not significant	7.18E-08	Model failed	Model failed	Model failed
1	262,407,194	262,416,861	Not significant	1.07E-07	5.39E-07	Not significant	1.24E-07
1	263,369,987	263,391,361	Not significant	1.04E-07	7.57E-07	Not significant	1.74E-07
1	263,421,836	263,469,212	Not significant	2.35E-07	Not significant	Not significant	2.57E-07
2	13,861,125	13,900,430	Not significant	1.37E-07	Not significant	Not significant	4.02E-07
2	13,963,629	14,005,045	Not significant	3.15E-07	Not significant	Not significant	1.01E-06
2	38,080,434	38,081,124	Not significant	3.18E-08	Model failed	Model failed	Model failed
2	58,153,317	58,183,581	Not significant	4.14E-07	Not significant	Not significant	4.83E-07
2	86,113,413	86,164,251	Not significant	2.76E-07	Not significant	Not significant	Not significant
2	86,356,070	86,702,015	Not significant	5.01E-09	2.37E-08	Not significant	5.05E-09
2	88,287,607	88,366,762	Not significant	5.63E-07	Not significant	Not significant	7.82E-07
2	136,485,236	136,485,296	Not significant	1.33E-07	6.55E-07	Not significant	4.94E-07
2	164,795,365	165,011,337	Not significant	4.54E-07	Not significant	Not significant	4.98E-07
3	20,227,521	20,376,157	Not significant	4.33E-07	Not significant	Not significant	Not significant
3	105,545,550	105,562,429	Not significant	5.61E-07	Not significant	Not significant	Not significant
3	134,593,364	134,952,658	Not significant	7.39E-09	4.02E-08	Not significant	8.67E-09
3	222,455,230	222,490,044	Not significant	7.59E-07	Not significant	Not significant	8.00E-07
3	222,503,713	222,513,147	Model failed	3.06E-07	Model failed	Model failed	Model failed
4	13,003,091	13,088,097	Not significant	3.61E-07	Not significant	Not significant	4.37E-07
4	16,637,915	16,645,702	Not significant	2.92E-08	Model failed	Model failed	Model failed
6	12,129,893	12,195,170	Not significant	3.36E-08	3.04E-07	Not significant	6.89E-08
6	19,545,275	19,571,212	Not significant	1.88E-09	8.61E-09	Not significant	4.18E-07
6	40,594,243	40,598,722	Not significant	3.78E-08	1.83E-07	Not significant	8.07E-08
6	52,709,410	52,878,546	Not significant	5.51E-07	Not significant	Not significant	Not significant
6	54,756,472	54,767,273	Not significant	6.03E-08	3.00E-07	Not significant	8.29E-08
6	55,360,902	55,466,024	Not significant	4.59E-07	Not significant	Not significant	8.26E-07
7	24,558	209,889	Not significant	3.60E-09	1.71E-08	Not significant	3.61E-09
7	8,344,719	8,375,455	Not significant	1.05E-07	4.85E-07	Not significant	1.11E-07

8	78,683,108	78,734,898	Not significant	2.03E-07	Not significant	Not significant	2.66E-07
9	57,985,117	58,057,584	Not significant	1.91E-07	Not significant	Not significant	2.79E-07
9	72,404,044	72,420,911	Model failed	2.89E-08	Model failed	Model failed	Model failed
9	73,438,286	73,446,206	Not significant	6.27E-07	Model failed	Model failed	Model failed
9	82,835,845	82,875,993	Model failed	Model failed	4.59E-09	Model failed	Model failed
10	18,080,744	18,096,127	Not significant	9.79E-08	4.20E-07	Not significant	9.23E-07
10	18,264,001	18,265,718	Not significant	1.58E-08	9.76E-08	Not significant	1.78E-07
10	18,306,871	18,342,967	Not significant	9.07E-07	Not significant	Not significant	Not significant
10	18,354,172	18,357,858	Not significant	7.05E-08	Model failed	Model failed	Model failed
10	29,520,015	29,535,013	Not significant	8.81E-08	Not significant	Not significant	2.89E-07
10	50,858,554	50,876,668	Not significant	1.67E-09	Model failed	Model failed	Model failed
10	63,549,495	63,585,886	Not significant	3.99E-08	Model failed	Model failed	Model failed
10	65,020,171	65,157,788	Not significant	2.17E-08	Model failed	Model failed	Model failed
10	65,167,961	65,227,127	Not significant	2.33E-07	Not significant	Not significant	2.68E-07
11	10,398,620	10,447,503	Not significant	3.08E-12	1.88E-11	Not significant	3.50E-12
12	31,886,985	32,003,794	Not significant	6.22E-07	Not significant	Not significant	9.03E-07
12	35,365,159	35,408,696	Not significant	7.18E-08	3.17E-07	Not significant	7.65E-08
13	8,224,592	8,239,443	Not significant	7.61E-08	4.04E-07	Not significant	9.23E-08
13	8,321,481	8,340,545	Not significant	1.41E-07	8.97E-07	Not significant	2.09E-07
13	14,324,927	14,540,728	Not significant	7.11E-10	3.37E-09	Not significant	2.57E-09
13	21,753,962	21,782,926	Not significant	2.47E-07	Not significant	Not significant	8.35E-07
14	29,320,129	29,404,807	Not significant	1.01E-07	4.48E-07	Not significant	1.02E-07
15	61,307,131	61,476,746	Not significant	2.91E-09	3.31E-08	Not significant	7.10E-09
17	18,436,762	18,537,277	Not significant	3.14E-07	Not significant	Not significant	5.34E-07
17	71,033,562	71,045,469	Not significant	2.90E-08	1.60E-07	Not significant	3.61E-08
17	71,176,215	71,176,575	Not significant	1.03E-07	Model failed	Model failed	Model failed
19	37,999,202	38,018,884	Not significant	6.86E-08	3.64E-07	Not significant	8.29E-08
20	14,097,841	14,118,776	Not significant	1.46E-07	Not significant	Not significant	2.51E-07
20	14,428,550	14,485,702	Not significant	2.23E-08	Model failed	Model failed	Model failed
21	45,263,507	45,345,862	Not significant	8.20E-07	Not significant	Not significant	Not significant
22	8,349,025	8,370,713	Not significant	1.67E-07	7.92E-07	Not significant	2.00E-07
22	8,379,465	8,400,165	Not significant	8.99E-08	Not significant	Not significant	4.89E-07
22	13,291,916	13,300,911	Not significant	8.31E-07	Not significant	Not significant	Not significant
22	44,452,770	44,548,081	Not significant	4.93E-07	Not significant	Not significant	Not significant
22	50,486,318	50,820,764	Not significant	3.50E-07	Not significant	Not significant	3.54E-07
23	39,064,510	39,116,473	Not significant	5.10E-08	2.27E-07	Not significant	1.60E-07
23	39,126,867	39,137,482	Not significant	2.35E-09	1.30E-08	Not significant	5.67E-09
23	39,387,808	39,419,963	Not significant	4.17E-07	Model failed	Model failed	Model failed
23	39,908,616	39,931,884	Not significant	2.04E-09	1.19E-08	Not significant	2.50E-09
23	40,052,504	40,140,303	Not significant	7.65E-09	1.45E-07	Not significant	3.21E-08
24	41,675,204	41,759,916	Not significant	1.02E-07	5.06E-07	Not significant	1.16E-07
25	4,586,431	4,883,217	Not significant	6.36E-07	Not significant	Not significant	6.61E-07

Supplementary Table 6 – Full list of haplotype blocks for which at least one RHM method significantly improved model fit for adult August weight. From left to right: chromosome, the haplotype block start (bp), haplotype block end (bp), and whether the inclusion of the regional GRM(s) significantly improved model fit (green), did not significantly improve model fit (yellow), or if a model failed to converge (red) for SNP-RHM, Hap-RHM, SNHap-RHM when compared to the null model, SNHap-RHM when compared to Hap-RHM, and SNHap-RHM when compared to SNP-RHM.

Chromosome	Block start (bp)	Block end (bp)	SNP-RHM	Hap-RHM	SNHap-RHM vs null	SNHap-RHM vs Hap-RHM	SNHap-RHM vs SNP-RHM
1	54,708,137	54,738,562	Not significant	1.15E-07	6.37E-07	Not significant	1.67E-07
6	67,126,647	67,275,637	Not significant	3.37E-08	1.62E-07	Not significant	3.62E-08
11	7,422,889	7,457,516	Not significant	2.96E-08	1.66E-07	Not significant	4.53E-08
12	1,315,546	1,498,521	Not significant	1.05E-08	4.93E-08	Not significant	1.07E-08
23	58,109,141	58,167,269	Not significant	9.55E-08	4.47E-07	Not significant	1.02E-07
26	13,997,630	14,095,506	Not significant	1.34E-07	6.11E-07	Not significant	1.41E-07

Supplementary Table 7 – Full list of haplotype blocks for which at least one RHM method significantly improved model fit for adult foreleg length. From left to right: chromosome, the haplotype block start (bp), haplotype block end (bp), and whether the inclusion of the regional GRM(s) significantly improved model fit (green) or did not significantly improve model fit (yellow) for SNP-RHM, Hap-RHM, SNHap-RHM when compared to the null model, SNHap-RHM when compared to Hap-RHM, and SNHap-RHM when compared to SNP-RHM.

Chromosome	Block start (bp)	Block end (bp)	SNP-RHM	Hap-RHM	SNHap-RHM vs null	SNHap-RHM vs Hap-RHM	SNHap-RHM vs SNP-RHM
3	120,620,484	121,115,743	Not significant	4.97E-08	2.42E-07	Not significant	5.44E-08
3	163,548,241	163,642,434	Not significant	2.23E-07	Not significant	Not significant	3.35E-07
3	163,752,532	163,836,906	Not significant	1.16E-10	1.11E-09	Not significant	2.21E-10
4	97,010,982	97,103,764	Not significant	1.07E-08	1.31E-07	Not significant	2.90E-08
6	40,627,521	40,814,530	Not significant	4.52E-07	Not significant	Not significant	6.96E-07
6	103,234,182	103,331,382	Model failed	6.09E-11	Model failed	Model failed	Model failed
7	7,380,870	7,489,218	Not significant	2.35E-10	1.24E-09	Not significant	2.47E-10
9	89,458,815	89,567,544	Not significant	5.82E-11	3.15E-10	Not significant	6.15E-11
11	7,422,889	7,457,516	Not significant	1.69E-12	1.03E-11	Not significant	3.49E-12
12	1,315,546	1,498,521	Not significant	2.11E-11	1.33E-10	Not significant	2.55E-11
12	68,397,546	68,481,603	Model failed	3.45E-10	Model failed	Model failed	Model failed
15	16,293,816	16,793,796	Not significant	1.89E-07	8.45E-07	Not significant	1.97E-07
16	**69,667,639**	**69,754,149**	**6.20E-07**	**Not significant**	**Not significant**	**Not significant**	**Not significant**
16	69,839,345	69,853,299	9.75E-07	1.55E-07	5.80E-07	Not significant	Not significant
16	70,412,884	70,442,751	9.52E-07	Not significant	Not significant	Not significant	Not significant
16	71,032,937	71,037,268	1.62E-07	5.47E-07	Model failed	Model failed	Model failed
17	17,198,504	17,283,637	Model failed	1.10E-10	Model failed	Model failed	Model failed
17	57,691,208	57,747,777	Not significant	5.18E-11	3.02E-10	Not significant	5.90E-11
17	57,758,713	57,776,113	Model failed	5.19E-11	Model failed	Model failed	Model failed
18	62,650,233	62,714,362	Not significant	7.51E-09	3.71E-08	Not significant	7.97E-09
20	358,178	802,423	Not significant	2.22E-07	9.51E-07	Not significant	2.30E-07
20	1,254,840	1,315,189	Not significant	1.41E-08	8.97E-08	Not significant	2.48E-08
20	47,667,243	47,726,425	Model failed	3.77E-09	Model failed	Model failed	Model failed
20	48,121,607	48,242,296	Not significant	3.90E-07	Not significant	Not significant	5.13E-07
21	13,200,687	13,254,516	Not significant	4.17E-11	Model failed	Model failed	Model failed
25	45,065,687	45,210,626	Not significant	2.45E-07	Not significant	Not significant	2.90E-07
26	15,931,720	15,994,925	Not significant	1.22E-07	5.97E-07	Not significant	1.38E-07

Supplementary Table 8 – Full list of haplotype blocks for which at least one RHM method significantly improved model fit for adult hindleg length. From left to right: chromosome, the haplotype block start (bp), haplotype block end (bp), and whether the inclusion of the regional GRM(s) significantly improved model fit (green), did not significantly improve model fit (yellow), or if a model failed to converge (red) for SNP-RHM, Hap-RHM, SNHap-RHM when compared to the null model, SNHap-RHM when compared to Hap-RHM, and SNHap-RHM when compared to SNP-RHM. Bold indicates that the block contains a top GWAS SNP for this trait.

Chromosome	Block start (bp)	Block end (bp)	SNP-RHM	Hap-RHM	SNHap-RHM vs null	SNHap-RHM vs Hap-RHM	SNHap-RHM vs SNP-RHM
16	68,687,286	68,705,421	5.45E-07	Not significant	Not significant	Not significant	Not significant
16	68,915,832	68,981,502	1.16E-10	8.41E-11	4.69E-11	Not significant	Not significant
16	69,129,929	69,138,953	2.22E-10	2.72E-09	2.46E-10	Not significant	Not significant
16	69,160,398	69,181,657	5.61E-10	2.20E-09	2.66E-09	Not significant	Not significant
16	69,226,093	69,306,236	2.62E-07	1.18E-08	5.63E-08	Not significant	Not significant
16	69,425,086	69,574,670	6.54E-11	4.37E-09	3.24E-10	Not significant	Not significant
16	69,667,639	69,754,149	1.28E-10	4.92E-07	Model failed	Model failed	Model failed
16	69,839,345	69,853,299	1.88E-10	2.91E-10	5.56E-10	Not significant	Not significant
16	69,856,342	69,943,027	4.66E-10	6.85E-09	1.53E-09	Not significant	Not significant
16	70,307,056	70,368,449	1.09E-08	2.09E-09	1.13E-08	Not significant	Not significant
16	70,412,884	70,442,751	4.14E-11	2.14E-10	1.51E-10	Not significant	Not significant
16	70,469,116	70,480,771	5.88E-12	4.08E-12	5.56E-11	Not significant	Not significant
16	70,492,788	70,861,290	2.60E-09	Not significant	1.34E-08	Not significant	Not significant
16	71,135,107	71,417,145	4.59E-09	Not significant	2.23E-08	Not significant	Not significant
16	71,538,789	71,603,934	1.22E-10	1.03E-10	Model failed	Model failed	Model failed
19	51,665,021	51,881,263	2.24E-07	Not significant	9.60E-07	Not significant	Not significant
19	51,908,871	52,340,442	2.48E-08	Not significant	1.12E-07	Not significant	Not significant
19	**52,376,602**	**52,543,759**	**4.35E-09**	**Not significant**	**2.05E-08**	**Not significant**	**Not significant**
19	52,733,279	52,798,829	2.34E-07	Not significant	1.00E-06	Not significant	Not significant

Supplementary Table 9 – Full list of haplotype blocks for which at least one RHM method significantly improved model fit for adult metacarpal length. From left to right: chromosome, the haplotype block start (bp), haplotype block end (bp), and whether the inclusion of the regional GRM(s) significantly improved model fit (green), did not significantly improve model fit (yellow), or if a model failed to converge (red) for SNP-RHM, Hap-RHM, SNHap-RHM when compared to the null model, SNHap-RHM when compared to Hap-RHM, and SNHap-RHM when compared to SNP-RHM. Bold indicates that the block contains a top GWAS SNP for this trait.

Chromosome	Block start (bp)	Block end (bp)	SNP-RHM	Hap-RHM	SNHap-RHM vs null	SNHap-RHM vs Hap-RHM	SNHap-RHM vs SNP-RHM
1	178,080,084	178,183,944	Not significant	8.89E-08	5.76E-07	Not significant	9.91E-07
3	125,238,635	125,340,391	Not significant	1.45E-08	1.21E-07	Not significant	2.69E-08
11	50,247,507	50,268,735	Not significant	8.73E-07	Model failed	Model failed	Model failed
18	36,416,557	36,442,672	Not significant	8.20E-07	Not significant	Not significant	Not significant
23	14,087,536	14,348,846	Not significant	9.61E-07	Not significant	Not significant	Not significant
23	21,076,868	21,139,467	Not significant	7.41E-07	Not significant	Not significant	8.18E-07

Supplementary Table 10 – Full list of haplotype blocks for which at least one RHM method significantly improved model fit for adult jaw length. From left to right: chromosome, the haplotype block start (bp), haplotype block end (bp), and whether the inclusion of the regional GRM(s) significantly improved model fit (green), did not significantly improve model fit (yellow), or if a model failed to converge (red) for SNP-RHM, Hap-RHM, SNHap-RHM when compared to the null model, SNHap-RHM when compared to Hap-RHM, and SNHap-RHM when compared to SNP-RHM.

Trait	Chromosome	Haplotype number	SNP	SNP position	Method of identification
Birth weight	1	3801	oar3_OAR1_175180452	175180452	GWAS
	2	1589	oar3_OAR2_81719138	81719138	Conditional analysis
	7	1173	oar3_OAR7_54963456	54963456	GWAS
Lamb foreleg	16	1368	oar3_OAR16_69873504	69873504	GWAS
Lamb hindleg	16	1368	oar3_OAR16_69873504	69873504	GWAS
Lamb metacarpal	3	1713	OAR3_100483326.1	94492563	GWAS
	16	1363	s22142.1	69679810	GWAS
	19	952	s74894.1	52470202	GWAS
Adult August weight	3	3696	oar3_OAR3_209859215	209859215	GWAS
	6	630	oar3_OAR6_35627634	35627634	GWAS
	9	847	oar3_OAR9_36632898	36632898	GWAS
Adult foreleg	7	1918	s48811.1	84579439	GWAS
	9	1165	oar3_OAR9_50469115	50469115	GWAS
	11	668	oar3_OAR11_30635038	30635038	GWAS
	16	1363	s22142.1	69679810	GWAS
	19	950	oar3_OAR19_52340442	52340442	GWAS
Adult hindleg	11	674	oar3_OAR11_30832740	30832740	GWAS
	16	1363	s22142.1	69679810	GWAS
	19	950	oar3_OAR19_52340442	52340442	GWAS
Adult metacarpal	11	681	oar3_OAR11_31212644	31212644	GWAS
	16	1393	oar3_OAR16_71129444	71129444	GWAS
	17	1291	oar3_OAR17_54802053	54802053	GWAS
	19	952	oar3_OAR19_52459313	52459313	GWAS
Adult jaw	2	*Falls between 2235 and 2236	oar3_OAR2_137162126	137162126	Conditional analysis
	20	1031	oar3_OAR20_46332617	46332617	GWAS

Supplementary Table 11 – Haplotype blocks containing the top GWAS SNPs from James et al. (2022). From left to right: trait, chromosome, haplotype block number, the SNP name, the SNP position, and whether the SNP was identified via GWAS or conditional analyses.

Supplementary Text

Across the whole sample of genotyped individuals, haplotype allele frequency ranged from 0.00005843 to 0.9895992, with 0.00005843 being the most commonly observed haplotype frequency (28.95% of haplotype alleles). A frequency of 0.00005843 equates to a haplotype allele being present on one chromosome in the entire sample. 75% of haplotype alleles had a frequency lower than 0.072 (present on less than 1232 chromosomes in the entire sample), and 90% had a frequency lower than 0.316 (present on less than 5408 chromosomes in the entire sample) (Figure 1). A breakdown of the number of SNPs, haplotype blocks and SNPs per block per chromosome can be found in Table 1.

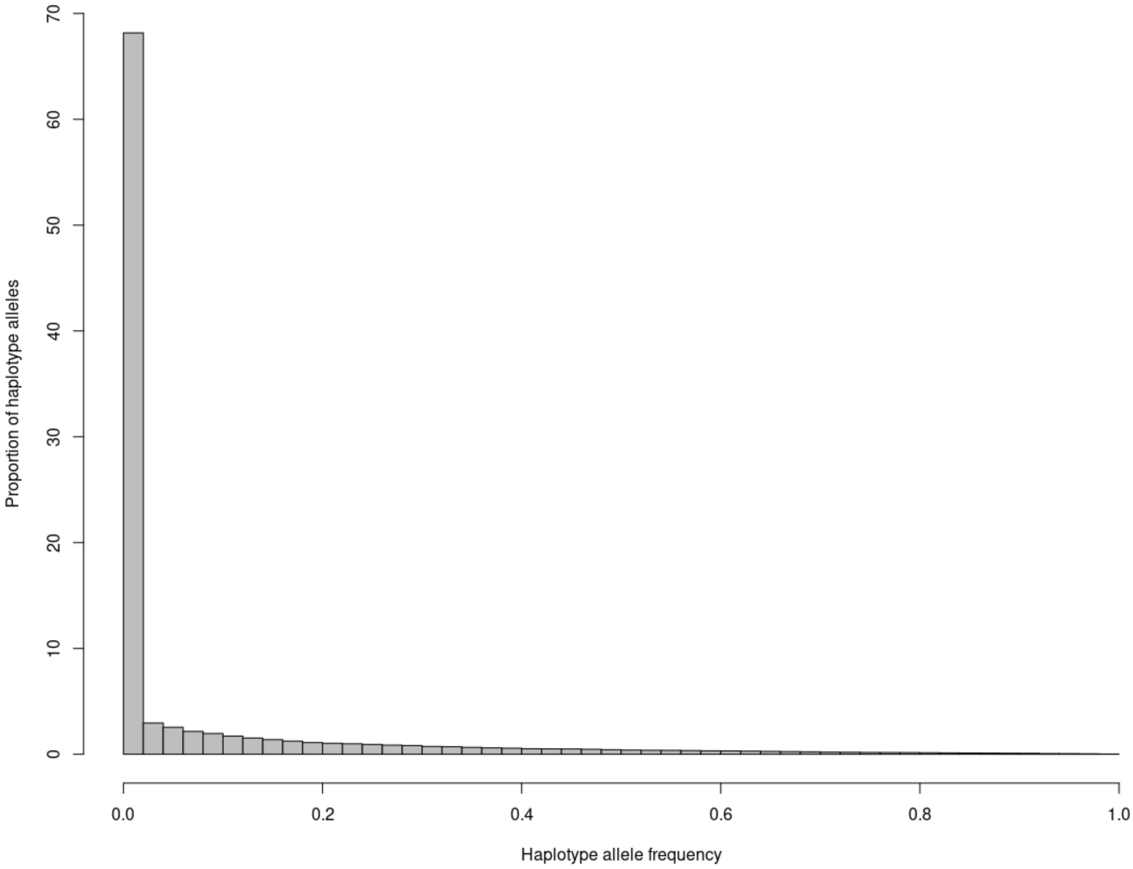

Figure 1: Proportion of haplotype alleles at each haplotype frequency over all regions.

Table 1 Number of SNPs per chromosome, number of haplotype blocks per chromosome, and average number of SNPs per haplotype block.

Chromosome	Number of SNPs	Number of haplotype blocks	Mean number of SNPs per haplotype block
1	47318	5466	8.66
2	41917	4426	9.47
3	38265	4081	9.38
4	20659	2259	9.15
5	18545	2132	8.70
6	19205	1908	10.07
7	17647	2267	7.78
8	15815	2029	7.79

9	16517	2177	7.59
10	15517	2223	6.98
11	11567	1371	8.44
12	13909	1665	8.35
13	13626	1427	9.55
14	10785	1205	8.95
15	13897	1537	9.04
16	11992	1400	8.57
17	12598	1694	7.44
18	11718	1357	8.64
19	10230	1058	9.67
20	9074	1115	8.14
21	7879	813	9.69
22	9352	1246	7.51
23	10004	1062	9.42
24	6362	492	12.93
25	7530	842	8.94
26	7353	873	8.42
